# Ultrasound nail assessment in patients with psoriasic arthritis: is there an association of findings with clinical scores?

**DOI:** 10.1186/s42358-024-00398-4

**Published:** 2024-09-27

**Authors:** Andrieli Caroline Mehl, Leonardo Michaelis Schmidt, Valderílio Feijó Azevedo

**Affiliations:** 1 The Federal University of Paraná, Curitiba, Parana Brazil; 2 Rheumatologist, Toledo, Parana Brazil; 3 Department of Internal Medicine, Hospital de Clínicas, Federal University of Paraná, Curitiba, Parana Brazil

**Keywords:** Psoriatic arthritis, Ultrasonography, Psoriasis nail

## Abstract

**Background:**

Psoriatic arthritis can involve several domains. Due to its multifaceted nature and its frequent comorbidities such as depression, obesity, osteoarthritis and fibromyalgia, it is difficult to monitor these patients because the clinical scores involve subjective data. High-resolution ultrasound probes allowed the evaluation of more superficial structures, such as the nails and their synovio-entheseal framework, in close relationship with the enthesis of the distal extensor digitorum tendon. Nail ultrasound studies vary in terms of the parameters and fingers studied and in their findings.

**Objectives:**

To describe the most significant sonographic nail changes and the most affected fingers in psoriatic arthritis and to verify the association of nail ultrasound findings with clinical scores (nail psoriasis severity index (NAPSI), ankylosing spondylitis disease activity score with C-reactive protein (ASDAS-CRP), minimal disease activity (MDA), disease activity index for psoriatic arthritis (DAPSA)).

**Methods:**

This was a cross-sectional study with 52 patients with psoriatic arthritis at the Hospital de Clínicas do Paraná and 50 controls. A total of 1016 nails were analyzed (517 from patients with psoriatic arthritis and 499 from controls). Ultrasonography of the nails of the 10 fingers was performed to assess the trilaminar appearance, measure the distance from the nail bed, identify synovitis of the distal interphalangeal joints and the presence of a power Doppler signal from the nail matrix/nail bed. The captured images were independently evaluated by a rheumatologist with expertise in musculoskeletal ultrasound. Data analysis was performed using IBM SPSS *Statistics* v.28.0.0 software, and the association of nail plate changes, nail bed distance and power Doppler signal with the NAPSI, DAPSA, MDA and ASDAS-PCR were calculated. *Spearman* correlation coefficients were estimated to analyze the correlations between pairs of quantitative variables. Student’s t test and the Mann‒Whitney U test were used to compare quantitative variables, and Fisher’s exact test was used to compare categorical variables between patients and controls. The nonparametric Mann‒Whitney U and Kruskal‒Wallis tests were used to compare groups according to the MDA or DAPSA classification.

**Results:**

The Doppler signal of the nail matrix and nail bed was more frequently identified in patients (44.2%) than in controls (6%), and the difference in the mean power Doppler signal between the two groups was significant (*p* < 0.001). Changes in the nail plate were more common in the right thumb (44.2%), left thumb (36.5%) and second finger on the right hand (32.7%). The number of fingers with nail plate changes, enthesitis, paratendinitis, grayscale synovitis and DIP involvement in the distal interphalangeal joints was higher among patients with psoriatic arthritis (*p* < 0.001). There were found some correlations between US findings and clinical scores: ultrasound nail involvement and the NAPSI score (*p* = 0.034), the number of fingers and mean change in the nail plate and the ASDAS-CRP (*p* = 0.030). DAPSA (remission/low activity versus moderate/high activity) was associated to the mean change in the nail plate (*p* < 0.013). CONCLUSIONS: Nail ultrasound has the potential to assist in the capturing of the actual disease activity status in patients with psoriatic arthritis.

**Supplementary Information:**

The online version contains supplementary material available at 10.1186/s42358-024-00398-4.

## Background

Psoriatic arthritis (PsA) belongs to the group of spondyloarthritis and is present in 7–30% of patients with psoriasis [1, 2]. According to its clinical manifestations, PsA is divided into five main domains that may or may not be combined: skin and nail disease, peripheral arthritis, axial disease, dactylitis and enthesitis [1, 3]. Patients with nail involvement are more predisposed to the development of arthritis, including arthritis of the distal interphalangeal joints (DIJs) [1–4]. Because it is a multifactorial disease, the definition of disease activity—used for patient follow-up and decision-making on whether to change or maintain treatment—with scores based on clinical and laboratory data is complex and imprecise [4, 5].

The onset of musculoskeletal involvement in PsA occurs in the synovio-entheseal complex, present in several anatomical locations [1, 6]. The extensor tendons of the fingers and the capsulo-ligamentous projections insert into the distal phalanges and are closely related to the base of the nail. Inflammatory processes that occur in the entheses extend to the tendons, joints, matrix and nail bed. Certain changes in the nail matrix and nail plate lead to thickening, yellowish spots, thimble depressions (pitting), undulations (crumbling), whitish spots (leukonychia), subungual hemorrhages, and detachment (onycholysis) and can be evaluated by the nail psoriasis severity index (NAPSI). Nail involvement in PsA interferes with the quality of life of patients [7, 8]; it occurs in 15–79% of patients with psoriasis while in 5–10% of cases, it is the only form of involvement. In psoriatic arthritis, nail involvement is even more common, occurring in 50–87% of patients [1–4].

Given the various phenotypes of psoriatic arthritis, it is difficult to find a single metric to evaluate and follow-up these patients. In addition to its multifaceted nature, psoriatic arthritis is also accompanied by multiple comorbidities, such as dyslipidemia, obesity, depression, and fibromyalgia, which can interfere both with the perception of disease activity by the patient and their physician and with the drug therapies that may be administered [4, 5, 9–11].

Ultrasonography (US) is an important diagnostic investigation method for rheumatologists [12–18]. With high-resolution transducers, nail US has been to be widely used to assist in the diagnosis, prognosis and treatment of diseases that affect the nail as it is able to identify minimal morphostructural changes when involvement is still subclinical [13, 18, 19]. US has been demonstrated to be beneficial in PsA, especially in the investigation of synovitis, tenosynovitis and enthesitis, which sometimes presents without evident clinical manifestations [6, 20–22]. The presence of sonographic changes may precede clinical nail and joint PsA manifestations [6, 15, 19, 23].

Nail evaluation is performed with high-frequency US ( > = 15 MHz) in B-mode to detail structures such as the usual trilaminar pattern of the nail and evaluate the nail matrix and nail bed. Power Doppler (PD) can identify changes in the microvasculature and can be used in the nail matrix and nail bed to quantify the presence of inflammation at the sites of enthesis [6, 15, 16, 19, 20, 23, 24].

With spectral Doppler, it is possible to quantify abnormal blood flow by calculating the vessel resistivity index. The lower this resistivity index is, the greater the association with inflamed neovessels or vessels; a value lower than 0.4 has been observed when an inflammatory process is present in the nail [25].

The earliest nail alterations that can be observed on US are those of the morphology of the ventral plate, which loses its hyperechoic definition and presents with focal irregularities with hyperechoic deposits [12, 15, 19, 25, 26].

In the more advanced stages of nail psoriasis, the trilaminar pattern is lost, and only a hypoechoic and thick lamina can be identified. Nail involvement in psoriasis, particularly the loss of its trilaminar appearance, is associated with an increased risk of psoriatic arthritis [13, 19].

When used as a test to help better define disease activity, ultrasound evaluation of the nails of patients with psoriatic arthritis, together with previously validated clinical scores, may help in making therapeutic decisions for this disease. It is still necessary to define which fingers and nail ultrasound parameters to consider, since there is great variability in the literature and none of the selections have been validated to date [12–16, 18]. Our study aims to contribute to the field by seeking to describe the most significant sonographic changes, the most affected fingers and whether there is an association between nail US findings and previously used clinical scores (NAPSI, ankylosing spondylitis disease activity score with C-reactive protein (ASDAS-CRP), minimal disease activity (MDA), and disease activity index for psoriatic arthritis (DAPSA).

## Methods

This cross-sectional study was approved by the Research Ethics Committee of the Hospital de Clínicas do Paraná, with Certificate of Ethical Assessment (CAAE**)** 57789516.0.0000.0096. A total of 102 participants (52 diagnosed with psoriatic arthritis and 50 in the control group) between March 2022 and January 2023 were included. The control group comprised the healthy companions of outpatients, medical students, a team of health professionals from the Hospital de Clínicas and employees/family members of a company providing park maintenance services. Participants in the psoriatic arthritis group needed to meet the Classification for Psoriatic Arthritis (CASPAR) criteria for Psoriatic Arthritis. Gout, rheumatoid arthritis, Behcet’s disease and other inflammatory joint or nail diseases were exclusion criteria. Nails with trauma or infection were excluded. All participants received and signed a consent form after having any concerns clarified by the researcher. Participants with psoriatic arthritis were consecutively selected in the order in which they presented for consultation at the Rheumatology outpatient clinic if they met the CASPAR and inclusion criteria without meeting any of the exclusion criteria.

### Data collection

The participants were evaluated in a dark room, seated with their hands resting on a stretcher or table. All examinations were performed by the same researcher, trained by a rheumatologist certified in ultrasound and with extensive experience in rheumatological ultrasound examination, who was also the independent reader of the study. All images were captured and sent to the independent reader who read and scored morphological lamina changes, enthesitis, paratendinitis, bed thickening, distal interphalangeal synovitis and Doppler scores of the bed and matrix.

The researcher was blinded to the demographic data and measurements taken at the consultation (NAPSI, psoriasis area and severity index (PASI), body surface area (BSA), MDA, DAPSA, Leeds enthesitis index (LEI) and ASDAS-CRP) until the end of the ultrasound examinations.

**Fig. 1:**
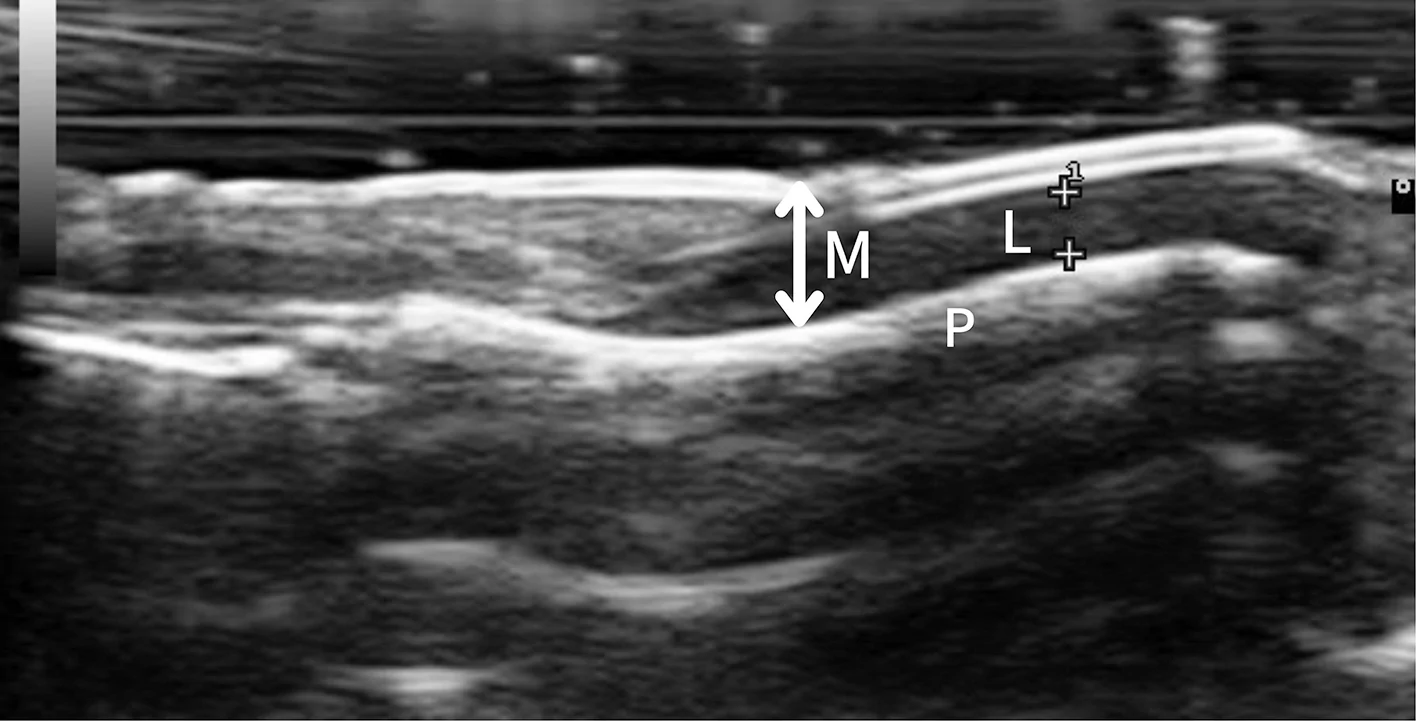
Ultrasound anatomy and nail measurements legend. L- nail bed; M- nail matrix, distance between + is the thickening of the nail bed (between the ventral nail plate: +1 and the periosteum of the distal phalanx: P)

The ultrasound devices used were an Esaote MyLab50X Vision (portable) and an Esaote MyLab40, both with similar software, with a high-resolution, 18 MHz LA435 linear transducer. A thick layer of gel was applied to each nail for better image performance. B-mode ultrasound was used to evaluate the trilaminar appearance of the nail in grayscale (GS) according to Wortsman et al. [18], the thickness of the nail bed—the distance between the ventral plate at the height of the middle third of the plate and the bone margin of the distal phalanx, grayscale synovitis in the distal interphalangeal joint, thickening of the extensor tendon, and heterogeneity and thickening of the tissue around the extensor tendon to detect paratendinitis. The findings about thickness are similar in matrix and bed nails, then we chose assess just one of them (Fig. 1).

The vascularity was assessed in nail and matrix because they are contiguous. Power Doppler imaging was used to determine any increased vascularization in the bed and matrix and to assess the distal interphalangeal joint to characterize synovitis and classify it according to Gutierrez et al. [27]. To quantify the flow, reduce artifacts and increase the specificity of the power Doppler findings in the nail matrix and nail bed, spectral Doppler imaging was used to calculate the vessel resistivity index when the power Doppler signal was positive. The score for each finger (**THE FINGER SCORE**) was also calculated as the sum of the number of points obtained from: finger GS (0–4), power Doppler signal of the bed and matrix (0–3), presence of enthesitis of the distal finger extensor (0–1), presence of paratendinitis of the DIJ (0–1), and degree of synovitis of the DIJ according to GS imaging (0–3) and power Doppler (0–3). For the scales 0–1: 0 not present and 1 present.

The classification of the nail plate used was from Wortsman (2004): 0 (normal trilaminar appearance), type 1 (focal hyperechoic involvement of the ventral plate without involvement of the dorsal plate), type 2 (loosening of the borders of the ventral plate with normal dorsal plate), type 3 (appearance of wavy plates—ventral and dorsal), type 4 (loss of definition on both plates) [18].

Semiquantitative nail classification using power doppler (PD) was in accord to Gutierrez (2012): PD 0 (no signal), PD 1 (confluent signal in less than 25% of the studied area), PD 2 (confluent signal between 25 and 50% of the studied area), PD 3 (confluent signal in more than 50% of the studied area) [27].

### Statistical analysis

The data were analyzed using IBM SPSS *Statistics* v.28.0.0. The results obtained in the study are described as the mean, standard deviation, median, minimum and maximum values (quantitative variables) or absolute frequency and percentage (categorical variables).

The associations between nail plate changes–means or scores—, nail bed distance, and power Doppler signal and the NAPSI, DAPSA and MDA were calculated.

Spearman’s correlation coefficients were estimated to analyze the correlations between pairs of quantitative variables. Comparisons between the patient and control groups in terms of quantitative variables were made using Student’s t test for independent samples or the nonparametric Mann‒Whitney U test. To compare groups defined by the MDA or DAPSA classifications, the nonparametric Mann‒Whitney U and Kruskal‒Wallis tests were used.

Regarding categorical variables, the groups were compared using Fisher’s exact test. For the variables evaluated by two examiners, the agreement between them was analyzed by estimating the *Kappa* coefficients of agreement (dummy variables) or the intraclass correlation coefficient (quantitative variables; mixed effects model, single measures and absolute agreement). Data from examiner 1 were otherwise considered throughout the analysis. P values < 0.05 indicated statistical significance.

## Results

The data from 52 participants with psoriatic arthritis (Study Group) and 50 participants without the disease (Control Group) were analyzed. A total of 1016 nails were analyzed, 517 from the psoriatic arthritis group and 499 from the controls; 4 nails were excluded due to trauma.

The results indicated a significant difference between the groups regarding age (*p* = 0.012); on average, the age of the patient group was 7 years older than that of the control group, 56.1 ± 12.2 (23–84) versus 49.0 ± 15.6 (22–77) years, respectively. The groups were homogeneous, with no significant differences between patients and controls regarding the percentages of individuals who were male (59.6% versus 48%), Caucasian (84.6% versus 92%) and manual laborers (35.4% versus 34%, respectively) (Table 1).

**Table 1:** Demographic characteristics of the control and study groups

Variable	Classification	Control group *N* (%)	Study (patients) group *N* (%)	P*
Age (years)	(mean ± standard deviation)	49.0 ± 15.6 (22–77)	56.1 ± 12.2 (23–84)	**0.012**
Sex	Male Female	24 (48) 26 (52)	31 (59.6) 21 (40.4)	0.321
Race	Nonwhite White	4 (8) 46 (92)	8 (15.4) 44 (84.6)	0.359
Manual labor	No Yes	33 (66) 17 (34)	31 (64.6) 17 (35.4)	1

*Student’s t test for independent samples (age); Fisher’s exact test (categorical variables); *p* < 0.05. Bold emphasis is statistical relevance

Among the participants in the psoriatic arthritis group, 57.7% had peripheral arthritis (and all these patients had distal interphalangeal involvement) without associated axial involvement (defined by the care team as an inflammatory axial clinical picture with or without image alteration). Among the 52 patients, all had a personal history of or current psoriasis, and the most common form was vulgaris (46.15%), which presented alone. There was no description of the type of psoriasis in the medical records of 2 patients. Only 1 patient had isolated nail psoriasis (1.92%), and in the other 17.31%, clinical involvement of the nail was associated with psoriasis vulgaris or scalp or inverted psoriasis (9 patients), as shown in Table 2.

**Table 2:** Descriptive characteristics of the group of patients

Classification	Total valid	*N* (%)
Axial involvement	52	
No		30 (57.7)
Yes		22 (42.3)
Family history	44	
No		32 (72.7)
Yes		12 (27.3)
HLA-B27	14	
No		11 (78.6)
Yes		3 (21.4)
Psoriasis	52	
Vulgar		24 (46.15)
Scalp		4 (7.69)
Nail Nail + vulgar or Nail + inverted Nail + vulgar + scalp		1 (1.92) 6 (11.54) 3 (5.77)
Others		12 (23.04)
No description		2 (3.84)

Regarding lifestyle, 54.2% of the patients with psoriatic arthritis described having a sedentary lifestyle, 12.2% had a history of previous smoking (≥ 3 months), and only 2% were current active smokers. Most psoriasis patients had at least one cardiovascular risk factor (82.4%), including arterial hypertension, dyslipidemia, obesity, previous cardiovascular events, and diabetes mellitus, the latter present in 31.4% of the patients. Regarding treatment, few patients were using constant-dose anti-inflammatory drugs (3 patients), while 62% were using conventional disease-modifying antirheumatic drugs (DMARDs), and 76.5% were using biological DMARDs; of these, 53.8% were using TNF-alpha inhibitors. Other clinical characteristics of the participants with psoriatic arthritis are presented in Table 3.

**Table 3:** Clinical features of participants with psoriatic arthritis

Features	Total valid	*N* (%)
Fibromyalgia	52	
No		48 (92.3)
Yes		4 (7.7)
Osteoarthritis	49	
No		26 (53.1)
Yes		23 (46.9)
Involvement of the DIP	49	
No		44 (89.8)
Yes		5 (10.2)
Uveitis	52	
No		47 (90.4)
Yes		5 (9.6)
Peripheral arthritis	52	
No		0 (0)
Yes		52 (100)
Enthesitis	50	
No		24 (48)
Yes		26 (52)
Dactylitis	49	
No		27 (55.1)
Yes		22 (44.9)
MDA	51	
No		32 (62.7)
Yes		19 (37.3)
LEI	50	
0		31 (62)
1		7 (14)
2		5 (10)
3		3 (6)
4		3 (6)
5		1 (2)
HAQ/HAQS	39	
< 0.5		20 (51.3)
0.5–1		14 (35.9)
> 1		5 (12.8)
Positive MRI	20	
No		17 (85)
Yes		3 (15)
Sacroiliitis X-ray	26	
No		15 (57.7)
Yes		11 (42.3)

*Notes*: DIP: distal interphalangeal, MDA = Minimun Disease Activity, LEI = Leeds enthesitis index; MRI = Magnetic resonance imaging, NSAIDS = Non-steroidal anti-inflammatory drugs, DMARDS = Disease modifying antirheumatic drugs

Regarding disease activity, 37.3% achieved remission according to the MDA score, while the mean and median DAPSA scores were 12.69 ± 13.11 (0.10–86.06) and 10, respectively, indicating low disease activity. Regarding cutaneous involvement, most patients had low scores, with a BSA 2.01% ± 3.18 (0–18) and PASI 1.74 ± 2.77 (0–13.70), while nail involvement was moderate according to the NAPSI (mean 16.10 ± 13.92 (0–50), median 13) (Supplementary Material).

In the individual evaluations for each finger, according to the Wortsman et al. [18] classification, the finger whose nail plate was most frequently affected was the right thumb (44.2%), followed by the left thumb (36.5%) and the second finger on the right hand (32.7%) (full data available in the Supplementary Material); for patients with psoriatic arthritis THE FINGER SCORE showed the most affected were the right thumb, left thumb, second and third fingers of the right hand (Table 4 and Graphic 1).

**Table 4:** Ultrasound score of each finger (quirodactyl) in patient group

Finger	Score				
	*n*	Mean	Median	Minimum	Maximum
1QDD	52	1,67	1	0	5
2QDD	52	0,87	0	0	5
3QDD	52	0,87	0	0	6
4QDD	52	0,62	0	0	5
5QDD	52	0,67	0	0	4
1QDE	52	1,13	1	0	6
2QDE	52	0,85	0	0	6
3QDE	52	0,67	0	0	4
4QDE	52	0,77	0	0	5
5QDE	52	0,71	0	0	5
Mean score	52	0,88	0,65	0	4,60

QD: quirodactyl

**Graphic 1:**
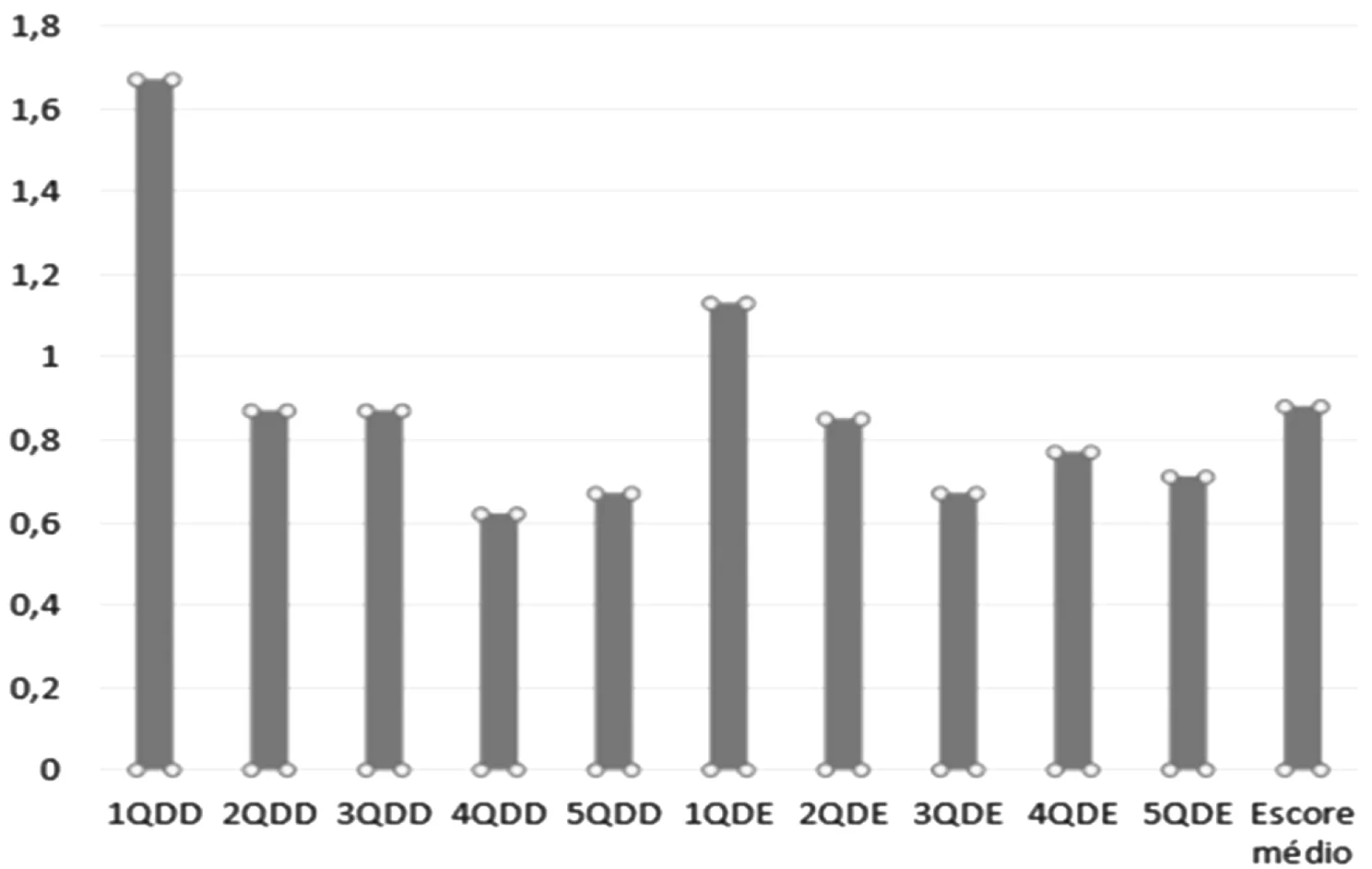
Average ultrasound score of each finger of patients in the psoriatic arthritis group. Legend: qd– quirodactyl (FINGER)

### Score analysis

#### Comparison of the group scores

The score for each finger was calculated as the sum of the number of points obtained in grayscale imaging according to Wortsman et al. [18] of the finger nail (FN), PD score of the bed and matrix, presence of enthesitis, presence of paratendinitis, and DIJ synovitis score according to grayscale imaging and power Doppler.

For the score of each finger and for the mean score of the 10 fingers, the results were different between groups, as shown in Table 5.

**Table 5:** Nail score of each finger and the mean score of the fingers

Score	Number	Mean	Median	Minimum	Maximum	*p* ^*^
1 QDD						
Control	50	0.10	0	0	2	
Patients	52	1.67	1	0	5	< 0.001
2 QDD						
Control	50	0.02	0	0	1	
Patients	52	0.87	0	0	5	< 0.001
3 QDD						
Control	50	0.00	0	0	0	
Patients	52	0.87	0	0	6	< 0.001
4 QDD						
Control	50	0.02	0	0	1	
Patients	52	0.62	0	0	5	< 0.001
5 QDD						
Control	50	0.00	0	0	0	
Patients	52	0.67	0	0	4	< 0.001
1 QDE						
Control	50	0.08	0	0	2	
Patients	52	1.13	1	0	6	< 0.001
2 QDE						
Control	50	0.00	0	0	0	
Patients	52	0.85	0	0	6	< 0.001
3 QDE						
Control	50	0.02	0	0	1	
Patients	52	0.67	0	0	4	< 0.001
4 QDE						
Control	50	0.00	0	0	0	
Patients	52	0.77	0	0	5	< 0.001
5 QDE						
Control	50	0.00	0	0	0	
Patients	52	0.71	0	0	5	< 0.001
Mean score of 10 fingers						
Control	50	0.02	0	0	0	
Patients	52	0.88	0.65	0	5	< 0.001

*Notes* QD: quirodactyl

* Nonparametric Mann‒Whitney U test, *p* < 0.05

#### Determination of a cutoff point for the mean score (ROC curve)

**Fig. 2:**
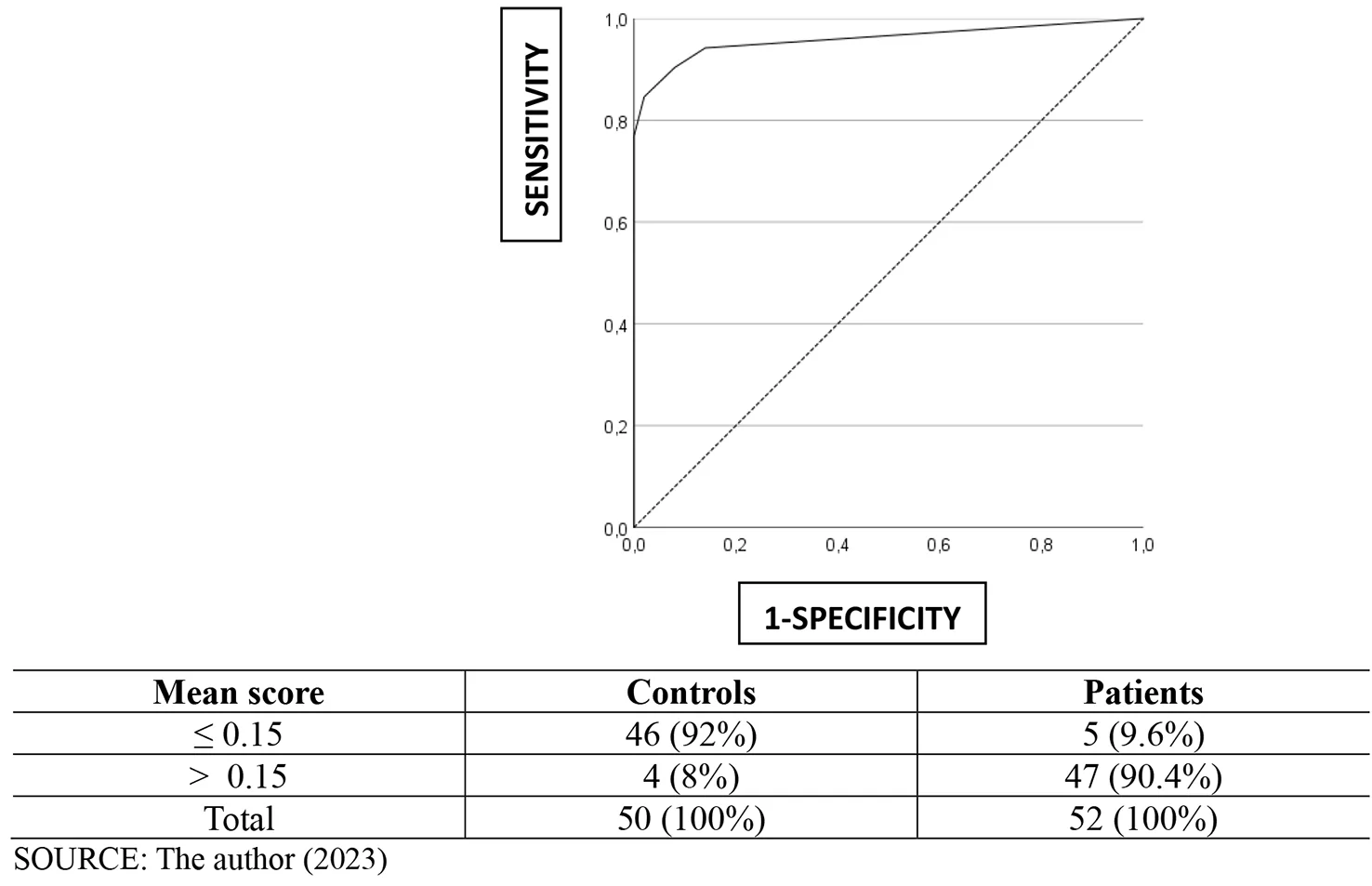
Roc curve for the presence of psoriatic arthritis. *Notes*

To determine a cutoff point for the mean score in identifying the disease, ROC curve analysis was performed. The area under the curve was equal to 0.96 (*p* < 0.001), indicating that the mean score differentiated between the disease and control groups well. The cutoff point obtained from curve fitting was equal to 0.15. Thus, mean score values > 0.15 correspond to the presence of the disease, and mean score values ≤ 0.15 correspond to the absence of the disease. The estimated sensitivity for this cutoff point is 90.4%, and the specificity is 92.0%, as shown in Fig. 2.

**Table:** 

Average score	Groups			
	Control		Study (patients)	
	*N*	%	*N*	%
≤ 0,15	46	92%	5	9,6%
> 0,15	4	8%	47	90,4%
Total	50	100%	52	100%

### Analysis of variables related to ultrasonography

#### Comparison of groups in relation to quantitative variables

**Fig. 3:**
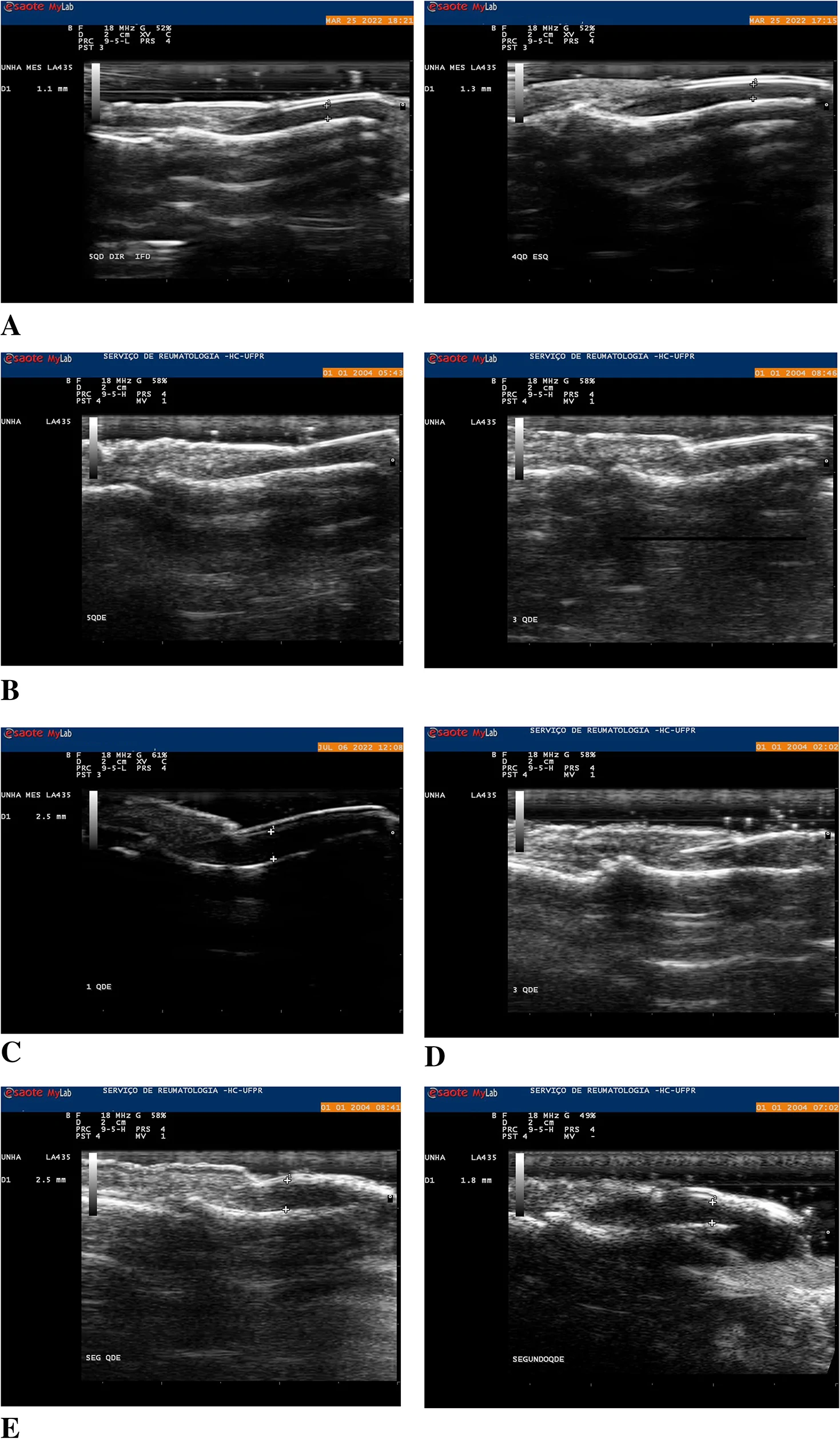
Images with changes in the nail plate according to Wortsman (2004). *Notes* (**A**) Grayscale (GS 0) and measuring of the distance from the nail bed with 1.1 mm and 1.3 mm, respectively. (**B**) Grayscale (GS 1). (**C**) Grayscale (GS 2) and measuring of the distance from the nail bed with 2.5 mm. (**D**) Grayscale (GS 3). (**E**) Grayscale (GS 4)

**Fig. 4:**
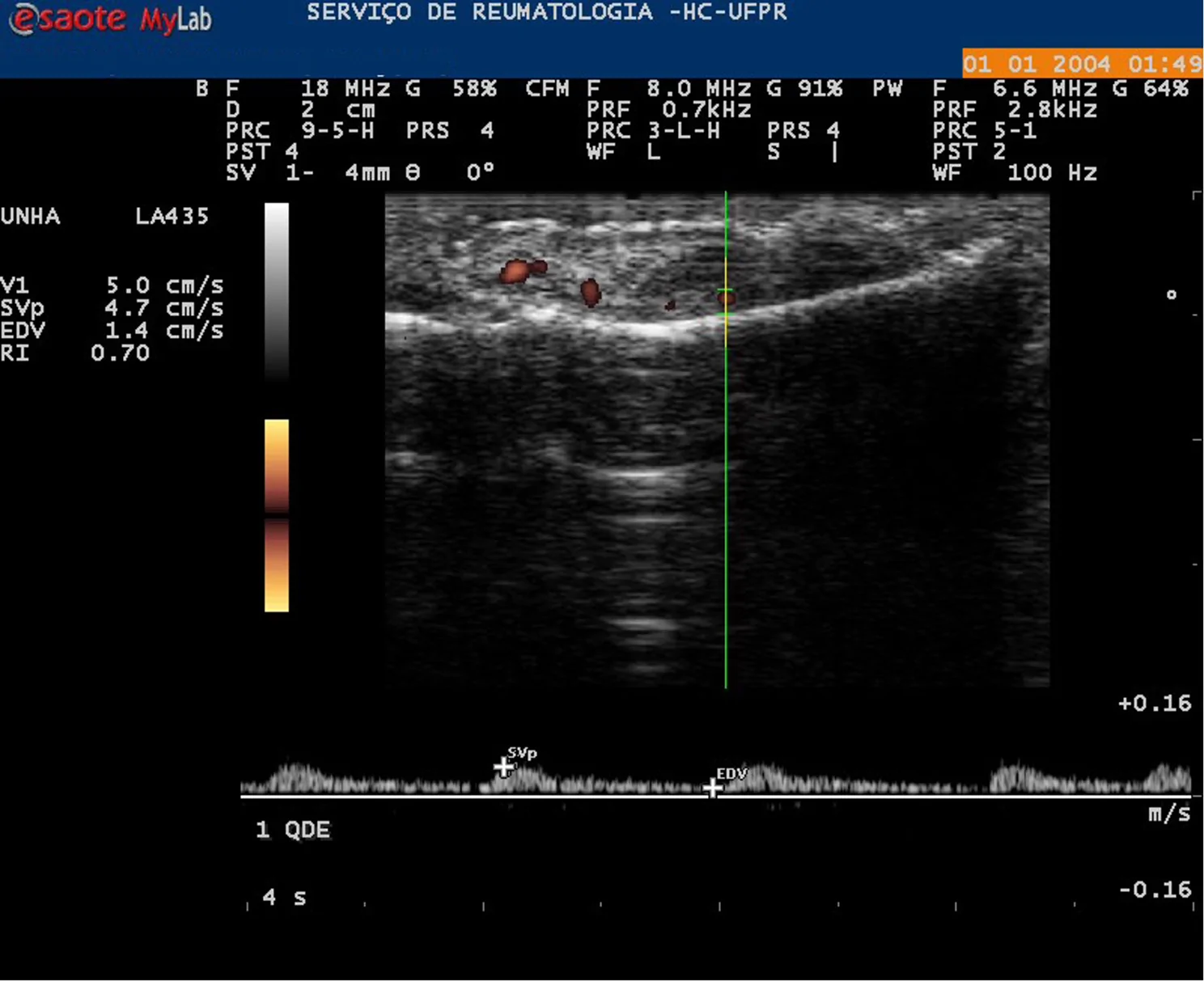
Power doppler imaging and calculation of the RI (resistivity index) of the nail bed. *Notes* Power doppler of the nail bed grade 1. Vessel resistivity index 0.70

The number of fingers showing grayscale changes according to the Wortsman et al. [18] classification, as shown in Fig. 3, was statistically higher in the patient group, both for major changes—grades 3 or 4—and for any grade. The number of fingers with enthesitis, paratendinitis or synovitis according to grayscale imaging was different between the two groups; participants with psoriatic arthritis had more affected fingers than the control participants. Participants with psoriatic arthritis also had a higher mean power Doppler signal in the nail bed and matrix compared to controls; an example positive power Doppler image shown in Fig. 4. On the other hand, the power Doppler signal of the joints and the distance from the nail bed (1.79 mm patients versus 1.67 mm control; *p* = 0.073) showed no differences between the groups (Table 6).

**Table 6:** Descriptive statistics of the variables according to the groups

Group	*N*	Mean	Median	*p* ^*^
Number of fingers with GS score 1, 2, 3, 4				
Control	50	0.10	0	
Patients	52	3.08	2.50	<** 0.001**
Number of fingers with GS score 3 or 4				
Control	50	0.00	0	
Patients	52	0.87	0	< **0.001**
Mean GS score				
Control	50	0.02	0	
Patients	52	0.62	0.40	< **0.001**
Mean nail bed distance				
Control	50	1.67	1.70	
Patients	52	1.79	1.80	0.073
Number of fingers with bed and matrix PD score > 0				
Control	50	0.06	0	
Patients	52	0.96	0.50	
Mean bed and matrix PD				
Control	50	0.01	0	
Patients	52	0.11	0.05	< **0.001**
Number of fingers with enthesitis				
Control	50	0	0	
Patients	52	0.50	0	<** 0.001**
Number of fingers with paratendinitis				
Control	50	0	0	
Patients	52	0.54	0	< **0.001**
Number of fingers with grayscale synovitis > 0				
Control	50	0	0	
Patients	52	0.48	0	< **0.001**
Mean grayscale synovitis				
Control	50	0	0	
Patients	52	0.06	0	< **0.001**
Number of fingers with PD synovitis > 0				
Control	50	0	0	
Patients	52	0	0	–
Mean PD synovitis				
Control	50	0	0	
Patients	52	0	0	–
Number of fingers with DIP involvement				
Control	50	0.58	0	
Patients	52	1.77	1.00	< **0.001**

*Notes* GS = grayscale; PD = power doppler

* Nonparametric Mann‒Whitney U test, *p* < 0.05. Bold emphasis is statistical relevance

The frequency of involvement according to the presence of a power Doppler signal of the bed and matrix was higher in the psoriatic arthritis group than in the control group (44.2% versus 6%), and none of the calculated resistivity index values were < 0.4 (available in the Supplementary Material). None of the fingers presented with synovitis on power Doppler in the distal interphalangeal joint in either group.

#### Comparison of categorical variables between groups

The groups were significantly different (*p* < 0.001) regarding the presence of nail plate alterations, enthesitis, paratendinitis, grayscale distal interphalangeal synovitis and the DIP involvement, all of which were more frequent in the group of psoriatic patients (Table 7).

**Table 7:** Descriptive statistics of the categorical variables according to the groups

Variables	Classification	Control *N* (%)	Patients *N* (%)	*p*
Any finger with GS 1,2,3 or 4?	No	45(90)	6 (11.5)	
	Yes	5 (10)	46 (88.5)	**< 0,001**
Any finger with GS 3 or 4?	No	47 (94)	26 (50)	
	Yes	3 (6)	26 (50)	**< 0,001**
Any finger with enthesitis?	No	50 (100)	39 (75)	
	Yes	0 (0)	13 (25)	**< 0,001**
Any finger with paratendinitis?	No	50 (100)	38 (73.1)	
	Yes	0 (0)	14 (26.9)	**< 0,001**
Any finger with grayscale distal interphalangeal synovitis > 0?	No	50 (100)	36 (69.2)	
	Yes	0 (0)	16 (30.8)	**< 0,001**
Any finger with PD distal interphalangeal synovitis > 0?	No	50 (100)	52 (100)	
	Yes	0 (0)	0	1
Any finger with DIP involvement?	No	39 (78)	22 (42.3)	
	Yes	11 (22)	30 (57.7)	**< 0,001**

*Fisher´s exact test, *p* < 0,05. Bold emphasis is statistical relevance

*Notes GS: gray scale; PD: power doppler; DIP: distal interphalangeal **(primary or secondary osteoarthritis)**

#### Association of MDA and DAPSA with the variables ultrasound

There was no difference for patients with and without MDA and the results of the nail ultrasound findings (available in Supplementary Material). For the analysis with classifications DAPSA: ≤ 4 (disease in remission), > 4 and ≤ 14 (low activity), > 14 and ≤ 28 (moderate activity) and > 28 (high activity) the findings showed that there was no difference between the groups. For the categorical variables, it was not possible to perform the statistical test due to the low frequencies of the findings (available in Supplementary Material).

If two classifications of DAPSA were considered: ≤ 14 (remission or low activity) and > 14 (moderate or high activity) significant differences were found between the groups in terms of the number of fingers with the highest degree of nail plate alteration (type 3 or 4), the average nail plate alteration of all the patient’s fingers and the average score of the 10 fingers. All these parameters were significantly higher among patients with DAPSA > 14 (Table 8). Fifteen patients had DAPSA corresponding to moderate or high activity, and 37 patients had DAPSA corresponding to remission or low activity.

**Table 8:** Descriptive statistics of the variables according to the dichotomous DAPSA groups

DAPSA	*N*	Mean	Median	*p* ^*^
Number of fingers with GS score 1, 2, 3, 4				
Remission/low	37	2.76	2	
Moderate/severe	15	3.87	3	0.052
**Number of fingers with GS score 3 or 4**				
**Remission/low**	**37**	**0.78**	**0**	
**Moderate/severe**	**15**	**1.07**	**1**	**0.030**
**Mean GS score**				
**Remission/low**	**37**	**0.57**	**0.30**	
**Moderate/severe**	**15**	**0.74**	**0.70**	**0.013**
Mean nail bed distance				
Remission/low	37	1.81	1.80	
Moderate/severe	15	1.75	1.79	0.984
Number of fingers with bed/matrix PD > 0				
Remission/low	37	0.86	0	
Moderate/severe	15	1.20	1	0.185
Mean bed and matrix PD				
Remission/low	37	0.10	0	
Moderate/severe	15	0.13	0.10	0.178
Number of fingers with enthesitis				
Remission/low	37	0.30	0	
Moderate/severe	15	1	0	0.704
Number of fingers with paratendinitis				
Remission/low	37	0.49	0	
Moderate/severe	15	0.67	0	0.617
Number of fingers with grayscale synovitis > 0				
Remission/low	37	0.51	0	
Moderate/severe	15	0.40	0	0.447
Mean grayscale synovitis				
Remission/low	37	0.06	0	
Moderate/severe	15	0.05	0	0.603
Number of fingers with PD synovitis > 0				
Remission/low	37	0	0	
Moderate/severe	15	0	0	–
Mean PD synovitis				
Remission/low	37	0	0	
Moderate/severe	15	0	0	–
Number of fingers with DIP involvement				
Remission/low	37	1.84	1	
Moderate/severe	15	1.60	1	0.780
**Mean score of the 10 fingers**				
**Remission/low**	**37**	**0.80**	**0.60**	
**Moderate/severe**	**15**	**1.09**	**1**	**0.008**

*Notes* GS = grayscale; PD = power doppler

* Nonparametric Mann‒Whitney U test, *p* < 0.05. Bold emphasis is statistical relevance

*Source* The Author

*Legends* Nº = number, d = digitum, GS = grayscale, PD = power doppler

Only a numerical difference was identified between the number of fingers with nail plate changes between patients with moderate and high DAPSA versus those with remission and low DAPSA. There was no significant difference in the presence of nail plate changes, enthesitis, paratendinitis, Doppler or grayscale DIJ synovitis, or DIP involvement between patients with remission/low DAPSA and those with moderate/high DAPSA. Due to the low frequency of cases, it was not possible to apply statistical tests (Supplementary Material).

### Correlation between quantitative clinical variables and quantitative us variables (restricted to patients)

In the evaluation between the clinical scores of activity in psoriatic arthritis versus the US quantitative variables of the nail, the NAPSI and ASDAS-CRP showed the highest estimated correlation coefficients (Tables 9 and 10).

**Table 9:** NAPSI and nail ultrasound findings. The degree of association can be classified as: excellent:|r| >0.90; Good|r| from 0.71 to 0.90; Moderate:|r| from 0.51 to 0.70; Weak|r| from 0.31 to 0.50 Adapted from Mukaka (2012)

Variables	*N*	Spearman’s correlation coefficient	*P*
NAPSI × No. of fingers with GS score 1,2,3,4	52	0,43	**0,002**
NAPSI × No. of fingers with GS score 3 or 4	52	0,45	**0,001**
NAPSI x Mean GS score	52	0,46	**0,001**
NAPSI x Mean nail bed distance	52	0,27	0,051
NAPSI x No. of fingers with PD > 0	52	0,28	**0,047**
NAPSI x Mean bed and matrix PD	52	0,27	0,052
NAPSI x Number of fingers with enthesitis	52	0,06	0,648
NAPSI × Number of fingers with paratendinitis	52	0,22	0,110
NAPSI x Number of fingers with grayscale synovitis > 0	52	0,02	0,881
NAPSI x Mean grayscale synovitis	52	0,03	0,805
NAPSI x Number of fingers with PD synovitis > 0	52	–	–
NAPSI x Mean PD synovitis	52	–	–
NAPSI x Number of fingers with DIP involvement	52	0,14	0,315
NAPSI x × Mean score of the 10 fingers	52	0,46	**0,001**

Notes: GS = Grayscale; PD = power doppler p<0,05 Bold emphasis is statistical relevance

**Table 10:** ASDAS-CRP and nail ultrasound findings. The degree of association can be classified as: excellent:|r| >0.90; Good|r| from 0.71 to 0.90; Moderate:|r| from 0.51 to 0.70; Weak|r| from 0.31 to 0.50 Adapted from Mukaka (2012)

Variables	*N*	Spearman’s correlation coefficient	*P*
ASDAS_PCR × No. of fingers with GS score 1,2,3,4	14	0.57	**0.034**
ASDAS_PCR × No. of fingers with GS score 3 or 4	14	0.59	**0.026**
ASDAS_CRP × Mean GS score	14	0.58	**0.030**
ASDAS_PCR × Mean nail bed distance	14	0.20	0.483
ASDAS_PCR × No. of fingers with PD > 0	14	0.29	0.316
ASDAS_CRP × Mean bed and matrix PD	14	0.29	0.308
ASDAS_CRP × Number of fingers with enthesitis	14	0.07	0.807
ASDAS_PCR × Number of fingers with paratendinitis	14	0.21	0.466
ASDAS_PCR × Number of fingers with grayscale synovitis > 0	14	−0.20	0.502
ASDAS_CRP × Mean grayscale synovitis	14	−0.17	0.550
ASDAS_PCR × Number of fingers with PD synovitis > 0	14	–	–
ASDAS_CRP × Mean PD synovitis	14	–	–
ASDAS_PCR × Number of fingers with DIP involvement	14	0.24	0.410
ASDAS_CRP × Mean score of the 10 fingers	14	0.66	**0.010**

Notes: GS = Grayscale; PD = power doppler p<0,05 Bold emphasis is statistical relevance

The NAPSI showed a weak correlation with the number of fingers with nail plate changes of any classification (1 to 4) or with major changes in the nail plate (grades 3 or 4), as well as with the means of the laminar change score for each patient or the mean of **THE FINGER SCORE** of the 10 fingers, which took into account nail plate alteration, presence of enthesitis in the distal digit extensor, presence of paratendinitis, grayscale and Doppler synovitis of the distal interphalangeal joint and power Doppler score for the nail bed or matrix as shown in Table 9.

The ASDAS-CRP showed a moderate correlation with the number of fingers with nail plate changes, the number of fingers with more severe nail plate changes (3 or 4) and the mean nail change, and the mean of **THE FINGER SCORE** of the 10 fingers as shown in Table 10.

The BSA and BASDAI showed no correlations with the nail ultrasound findings. The other correlations found were a weak correlation between DAPSA and the number of fingers with more severe alterations (grades 3 or 4) of the nail plate (Spearman correlation coefficient 0,3; *p* = 0,032), and between the PASI and the number of fingers with paratendinitis (Spearman correlation coefficient 0,41; *p* = 0,003). The corresponding tables and graphics are found in the Supplementary Material.

### Analysis of the agreement of the two examiners

#### Agreement between the two examiners in the presence of a finger with GS grade 1, 2, 3, 4, PD > 0, enthesitis, paratendinitis, synovitis, grayscale > 0, DIP involvement (binary variables) and quantitative variables related to US

This analysis was not performed for power Doppler synovitis because no patient or control presented with the condition. To assess the level of agreement of the two examiners, *kappa* coefficients of agreement were estimated for the binary variables, and intraclass correlation coefficients (ICCs) for the quantitative variables, and the 95% confidence intervals (95% CI) were calculated. The results were good for both of them (kappa > = 0.85 and ICC > = 0.904) (Supplementary Material).

## Discussion

One-third of psoriasis patients develop psoriatic arthritis (PsA). If nail psoriasis is present, the risk triples, particularly if onycholysis occurs, and is linked to distal inter-phalangeal arthritis [28].

In psoriatic arthritis, the main process is enthesitic and periarticular and not necessarily associated with synovitis [1, 6]. This complicates the clinical evaluation and potentially delays diagnosis. Ultrasound has proven valuable in managing these patients.

Most studies that evaluated the distance (or thickening) of the nail bed found a significant difference between patients with psoriasis/psoriatic arthritis and controls [12, 15, 16, 18, 25]. Gutierrez-Manjarrez et al. [19] identified normal measurements for the nail bed up to 2.5–3 mm after comparing patients with psoriatic arthritis and controls, while Sandobal et al. [16] found that a 2 mm nail bed could discriminate patients with psoriasis or psoriatic arthritis from healthy controls or controls with rheumatoid arthritis. In the present study, we did not find such a difference in nail bed thickness between patients with psoriatic arthritis and controls (1.79 versus 1.67 mm; *p* = 0.073), in agreement with the findings of De Rossi et al. [14]. This finding can be associated to the age difference between the groups in this study, in which, on average, the age of the patient group was 7 years older than that of the control and this could be associated to decreasing of nail bed distance. The age difference also can be implicated to the higher prevalence of distal interphalangeal involvement in psoriatic group, and this can be associated to the disease or primary osteoarthritis.

Nail ultrasound studies vary in the number of fingers examined, leading to different results. Regarding the number of fingers examined, studies have reported values ranging from 2 fingers—corresponding to the nail most clinically affected and that of the contralateral finger [13, 18] —or 3 fingers of the dominant hand plus fingers with clinical onychopathy or pain in the DIJs [16], or second and third fingers bilaterally [14], up to 10 fingers [15] or 12 digits (the 10 fingers of the hands and the 2 halluces) in the ultrasound evaluations [12]. Given the variability of the results in the literature, we chose to evaluate all fingers and verify if there were differences among the fingers for this study population according to the parameters evaluated. The finger most affected was the right thumb (44.2%), followed by the left thumb—both also identified by Wortsman et al. [18] with regard to nail plate changes—and regard to the mean FINGER SCORE the right thumb was also the most affected, followed by the left thumb, second and third fingers of the right hand. According to Mondal et al. [15], who also evaluated the 10 fingers, the right thumb was also the finger that most frequently presented with Wortsman’s alterations (93.33%). Acer Kasman et al. [12], who evaluated 12 digits (including the 2 halluces), found that the most discriminative fingers varied according to the parameter, each of which was evaluated individually: the left thumb for the nail plate index, the fifth finger of the left hand for nail bed and total thickening, and the third finger of the right hand for Doppler activity; in contrast, Mendonça et al. [25] found that the second and third fingers had the greatest changes on spectral Doppler and the lowest resistivity index, respectively.

To assess inflammation of the nail matrix and bed, in addition to measuring the thickness of these structures, local vascularization can also be analyzed using power Doppler imaging according to the grading proposed by Gutierrez-Manjarrez et al. [19] and quantified by measuring the nail vessel resistivity index (RI) by Doppler spectral imaging. Both power Doppler and the RI calculated by spectral Doppler are related to the neovascularization of inflammatory processes. Studies that evaluated the vessel RI in the nail matrix achieved divergent results. Among patients with psoriasis, both greater [29, 30] and sometimes lower values have been obtained with respect to controls [31]; among patients with psoriatic arthritis, the vessel RI is sometimes lower than in control patients [25]; and in a study comparing patients with psoriasis, psoriatic arthritis and controls, no difference was identified between groups [14]. Regarding the resistivity index, assessed in participants with positive power Doppler findings, no patient had an RI < 0.4. The vessel resistance index is still inconclusive in the evaluation of these patients. We believe that because it is measured from microvessels, the difference in Doppler sensitivity in different machines may interfere with the results.

Power Doppler signal at the nail bed and matrix was more frequent in psoriatic patients (44.2%) compared to controls (6%), and the mean score according to the Gutierrez et al. [28] classification was also significantly different—mean power Doppler 0.11 ± 0.05 (0-0.67) for the group with psoriatic arthritis versus 0.01 ± 0 (0-0.10) (*p* < 0.001) for the control group. This finding is in agreement with what Kasman et al. [12], Arbault et al. [26] and Sandobal et al. [16] found, while De Rossi et al. [14] and Mendonça et al. [25] found no difference.

For distal interphalangeal joints, grayscale synovitis findings differed between groups, but no power Doppler signal was found in any participant. We considered this finding related to the fact that most patients were on biological medications, circumstances under which the Doppler findings are the most rapidly modified. Positive power Doppler of the distal interphalangeal joint as a discriminative finding is not always investigated because there tends to be greater focus on bed/matrix and nail enthesis, but Sandobal et al. [16] and Arbault et al. [26], found differences between psoriatic and control individuals.

The association between ultrasound nail involvement and disease activity varies in the literature, both with regard to the scores compared and to the findings. Nail ultrasound identified more changes in psoriatic patients, even in clinically normal nails [13, 15, 16, 26]. Although these changes were more frequent when the nails were clinically affected, i.e., there is a good association with the clinical nail data verified by the NAPSI or modified NAPSI [13, 15, 32]. We also identified a weak but significant correlation between clinical nail involvement as verified by NAPSI and ultrasound alterations of the nail plate according to the Wortsman et al. [18] classification, both for the presence of this finding and for the mean severity of change.

While Mondal et al. [15] found a moderate correlation between the NAPSI and nail matrix thickness, our data did not support this. However, we identified a correlation between the mean **FINGER SCORE** of the 10 fingers—which considers abnormal nail plates, the presence of enthesitis of the distal finger extensor, the presence of paratendinitis, grayscale and power Doppler synovitis of the distal interphalangeal joint, and positive power Doppler of the bed or nail matrix–and the NAPSI.

There is a known positive relationship between nail psoriasis and more severe cutaneous psoriasis with a higher risk of psoriatic arthritis. We found a moderate correlation between the cutaneous involvement as calculated by the PASI and the number of fingers with paratendinitis. This is not the first report of a correlation between the severity of cutaneous disease and ultrasound findings of the nails. Mondal et al. [15] previously identified a correlation between the PASI and nail matrix thickness. No correlation was found between BSA and nail findings on US.

Regarding the composite indices, we did not find a correlation between MDA values and the ultrasound findings, but a correlation was identified with the DAPSA, unlike Mondal et al. [15], who found no such correlation.

When the patients were grouped into DAPSA ≤ 14 (remission or low activity) or > 14 (moderate or high activity), we found significant differences; specifically whith DAPSA > 14, patients were more likely to have fingers with greater involvement of the nail plate (grades 3 or 4), greater mean changes in the nail plate and greater mean scores of the 10 fingers. We also identified a weak correlation between DAPSA values and the number of fingers with more severe alterations (grades 3 or 4) of the nail plate. We found no association between MDA values and ultrasound findings.

While Arbault et al. [26] found no correlation between disease activity by ASDAS-CRP or CRP alone and any ultrasound parameters in patients with psoriatic arthritis, we found a moderate correlation between the ASDAS-CRP and the number of fingers with nail plate changes, the number of fingers with more severe nail plate changes (3 or 4), the mean of this nail change, and the mean score of the 10 fingers, but no correlation with BASDAI was found [1].

## Conclusions

Commonly used evaluation scores for psoriatic arthritis may not capture the actual activity status of the patient. Ultrasound shows to be a valuable tool for assessing nail changes in psoriatic arthritis, revealing significant differences and correlations with disease activity.

We showed that the thumbs and second and third fingers of the right hand are the most discriminating for evaluating psoriatic arthritis and could be considered in studies correlating ultrasound findings with later-stage disease activity. Nevertheless, we were able to show that the fingers most subjected to microtrauma (thumbs and right second finger) are the ones most often affected, which corroborates the Koebner phenomenon for psoriatic arthritis.

We found an association between ultrasound findings regarding changes in the nail plate and the distal interphalangeal joints (grayscale synovitis), as in previous studies, but we did not find changes with regard to bed thickening or changes in the power Doppler signal. These findings may be due to the Doppler resolution of the machine used and the fact that the duration of treatment with biological agents and the degree of activity of the patients in this sample, since inflammatory changes (which are primarily related to the Doppler signal) are the first to resolve.

The correlation between ASDAS-CRP and nail plate changes agrees with what is known about greater nail involvement in patients with more severe psoriatic disease. Axial involvement is known to be more common in longer-standing and more severe psoriatic arthritis.

The present study has limitations. The number of participants was small and more associations could be detected with larger samples, optimizing future findings to aid rheumatologist´s practice. Because this study involved imaging and data resulting from data collection, some of the data were missing, and some demographic variables and scores were not described for all patients. Furthermore, the ideal method for comparing ultrasound findings is to perform the exam in 2 stages, one for each examiner separately, which was not possible for this project but may be a strategy for future validation.

Expectedly, when evaluating all the fingers, the examination time was longer, which reduced the number of people willing to participate in the study. On the other hand, we were able to show that the fingers most subjected to microtrauma were the ones most often affected, which corroborates the Koebner phenomenon for psoriatic arthritis.

## Electronic supplementary material

Below is the link to the electronic supplementary material.

Supplementary Material 1

## Data Availability

The data that support the findings of this study are available from the corresponding author, upon reasonable request.
