# Supplementary material for: Ultrasound nail assessment in patients with psoriasic arthritis: is there an association of findings with clinical scores?
Source: Adv Rheumatol. 2024 Sep 27;64:75. doi: 10.1186/s42358-024-00398-4 (PMC13488798; doi:10.1186/s42358-024-00398-4)
Supplement: Supplementary file 1 [file 42358_2024_398_MOESM1_ESM.docx]

APPENDIX A- Metrics of patients

**Table 1.** Metrics of patients with psoriatic artrhritis

| **Variable** | **n** | **Mean** | **Standard deviation** | **Median** | **Minimum** | **Maximum** |
| --- | --- | --- | --- | --- | --- | --- |
| BASDAI | 18 | 4,18 | 2,61 | 3,40 | 0 | 9,60 |
| ASDAS_PCR | 14 | 2,77 | 1,08 | 2,95 | 0,90 | 4,11 |
| NAPSI | 52 | 16,10 | 13,92 | 13,00 | 0 | 50,00 |
| DAPSA | 52 | 12,69 | 13,11 | 10,00 | 0,10 | 86,06 |
| BSA | 51 | 2,01 | 3,18 | 1,00 | 0 | 18,00 |
| PASI | 51 | 1,74 | 2,77 | 0,60 | 0 | 13,70 |
| PCR | 52 | 0,50 | 0,63 | 0,32 | 0,03 | 3,48 |
|  |  |  |  |  |  |  |

APPENDIX B- RESULT OF THE NAIL PLATE

1- Involvement of the nail plate of each finger according to the Worstman classification (2004)

(To be continued)

| **Patient** | **gsQD1** | **gsQD2** | **gsQD3** | **gsQD4** | **gsQD5** | **gsQD6** | **gsQD7** | **gsQD8** | **gsQD9** | **gsQD10** |
| --- | --- | --- | --- | --- | --- | --- | --- | --- | --- | --- |
| 1 | 1 | 0 | 0 | 0 | 0 | 0 | 0 | 2 | 0 | 0 |
| 2 | 0 | 0 | 0 | 0 | 1 | 0 | 0 | 0 | 0 | 0 |
| 3 | 0 | 0 | 3 | 1 | 0 | 0 | 0 | 0 | 0 | 0 |
| 4 | 0 | 0 | 0 | 0 | 0 | 0 | 0 | 3 | 0 | 0 |
| 5 | 0 | 0 | 0 | 0 | 0 | 0 | 0 | 0 | 0 | 0 |
| 6 | 2 | 4 | 3 | 3 | 3 | 3 | 3 | . | 2 | 4 |
| 7 | 0 | 0 | 2 | 2 | 2 | 0 | 0 | 2 | 4 | 1 |
| 8 | 0 | 0 | 2 | 0 | 0 | 0 | 4 | 0 | 0 | 3 |
| 9 | 3 | 0 | 0 | 0 | 0 | 0 | 0 | 0 | 0 | 0 |
| 10 | 2 | 0 | 0 | 0 | 2 | 0 | 0 | 0 | 0 | 2 |
| 11 | 0 | 0 | 0 | 0 | 1 | 0 | 1 | 1 | 0 | 0 |
| 12 | 0 | 0 | 0 | 0 | 0 | 2 | 3 | 2 | 0 | 0 |
| 13 | 1 | 0 | 0 | 0 | 0 | 0 | 0 | 0 | 0 | 0 |
| 14 | 0 | 1 | 1 | 1 | 0 | 1 | 1 | 1 | 1 | 0 |
| 15 | 0 | 0 | 0 | 0 | 0 | 2 | 2 | 0 | 0 | 0 |
| 16 | 1 | 1 | 1 | 0 | 0 | 1 | 2 | 0 | 1 | 1 |
| 17 | 2 | 2 | 0 | 0 | 0 | 2 | 0 | 0 | 0 | 0 |
| 18 | 0 | 0 | 0 | 0 | 2 | 0 | 0 | 0 | 3 | 0 |
| 19 | 1 | 1 | 0 | 0 | 1 | 1 | 0 | 0 | 0 | 0 |
| 20 | 4 | 4 | 4 | 4 | 3 | 3 | 4 | 3 | 4 | 4 |
| 21 | 1 | 0 | 0 | 0 | 0 | 0 | 0 | 0 | 1 | 0 |
| 22 | 2 | 0 | 1 | 0 | 0 | 2 | 0 | 1 | 1 | 1 |
| 23 | 0 | 0 | 0 | 0 | 0 | 0 | 0 | 0 | 0 | 0 |
| 24 | 3 | 1 | 0 | 0 | 0 | 3 | 1 | 2 | 0 | 0 |
| 25 | 0 | 2 | 0 | 0 | 0 | 0 | 2 | 0 | 0 | 0 |
| 26 | 0 | 1 | 0 | 0 | 0 | 0 | 0 | 0 | 0 | 0 |
| 27 | 0 | 2 | 0 | 0 | 0 | 2 | 1 | 0 | 2 | 0 |
| 28 | 2 | 0 | 0 | 2 | 0 | 0 | 2 | 0 | 0 | 1 |
| 29 | 2 | 0 | 0 | 0 | 2 | 2 | 0 | 0 | 0 | 2 |
| 30 | 0 | 0 | 0 | 0 | 0 | 0 | 2 | 0 | 0 | 0 |
| 31 | 2 | 0 | 0 | 0 | 3 | 2 | 0 | 0 | 0 | . |
| 32 | 2 | 0 | 0 | 3 | 0 | 0 | 0 | 0 | 0 | 0 |
| 33 | 0 | 1 | 0 | 0 | 0 | 0 | 1 | 0 | 2 | 3 |
| 34 | 2 | 0 | 0 | 0 | 0 | . | 0 | 1 | 0 | 0 |
| 35 | 0 | 0 | 1 | 0 | 0 | 0 | 0 | 0 | 0 | 1 |

1- Involvement of the nail plate of each finger according to the Worstman classification (2004)

(conclusion)

| 36 | 0 | 0 | 0 | 0 | 0 | 0 | 0 | 0 | | 0 | 0 |
| --- | --- | --- | --- | --- | --- | --- | --- | --- | --- | --- | --- |
| 37 | 0 | 0 | 0 | 2 | 0 | 0 | 0 | 0 | | 0 | 0 |
| 38 | 0 | 0 | 0 | 1 | 0 | 0 | 0 | 0 | | 3 | 1 |
| 39 | 2 | 2 | 0 | 0 | 1 | 1 | 2 | 0 | | 0 | 0 |
| 40 | 2 | 1 | 2 | 0 | 0 | 2 | 0 | 3 | 0 | | 0 |
| 41 | 2 | 3 | 2 | 3 | 0 | 3 | 0 | 2 | 2 | | 1 |
| 42 | 0 | 0 | 0 | 0 | 0 | 0 | 0 | 0 | 0 | | 0 |
| 43 | 0 | 0 | 0 | 0 | 0 | 1 | 0 | 0 | 0 | | 0 |
| 44 | 0 | 0 | 1 | 0 | 0 | 0 | 0 | 1 | 0 | | 0 |
| 45 | 2 | 0 | 0 | 0 | 0 | 0 | 0 | 0 | 0 | | 0 |
| 46 | 0 | 0 | 1 | 0 | 1 | 0 | 0 | 0 | 2 | | 0 |
| 47 | 0 | 3 | 0 | 0 | 0 | 0 | 0 | 0 | 1 | | 1 |
| 48 | 0 | 0 | 0 | 0 | 0 | 2 | 0 | 0 | 0 | | 0 |
| 49 | 0 | 0 | 0 | 0 | 0 | 0 | 0 | 0 | 0 | | 0 |
| 50 | 2 | 3 | 1 | 0 | 4 | 3 | 4 | 1 | 1 | | 3 |
| 51 | 3 | 3 | 3 | 1 | 0 | 0 | 3 | 0 | 1 | | 0 |
| 52 | 0 | 0 | 0 | 0 | 0 | 0 | 0 | 0 | 0 | | 0 |

Notes: GS: gray scale; QD= quirodactyl

Source: The author (2023).

APPENDIX C –POWER DOPPLER RESULTS

1- Power doppler score of the bed and nail matrix of all participants

| **Participant** | **PD1qdd** | **PD2qdd** | **PD3qdd** | **PD4qdd** | **PD5qdd** | **PD1qde** | **PD2qde** | **PD3qde** | **PD4qde** | **PD5qde** |
| --- | --- | --- | --- | --- | --- | --- | --- | --- | --- | --- |
| 1 | 0 | 0 | 0 | 0 | 0 | 0 | 0 | 0 | 0 | 0 |
| 2 | 0 | 0 | 0 | 0 | 0 | 0 | 0 | 0 | 0 | 0 |
| 3 | 1 | 0 | 0 | 0 | 0 | 0 | 0 | 0 | 0 | 0 |
| 4 | 1 | 1 | 0 | 0 | 0 | 1 | 0 | 0 | 0 | 0 |
| 5 | 0 | 0 | 0 | 0 | 0 | 0 | 0 | 0 | 0 | 0 |
| 6 | 1 | 0 | 1 | 0 | 0 | 2 | 0 | . | 2 | 0 |
| 7 | 0 | 0 | 0 | 0 | 0 | 0 | 0 | 0 | 0 | 0 |
| 8 | 1 | 0 | 0 | 0 | 0 | 0 | 0 | 0 | 0 | 0 |
| 9 | 1 | 0 | 0 | 0 | 0 | 0 | 0 | 0 | 0 | 0 |
| 10 | 0 | 0 | 0 | 0 | 0 | 0 | 0 | 0 | 0 | 0 |
| 11 | 0 | 0 | 0 | 0 | 0 | 0 | 0 | 0 | 0 | 0 |
| 12 | 2 | 0 | 0 | 0 | 0 | 1 | 0 | 0 | 0 | 1 |
| 13 | 0 | 0 | 0 | 0 | 0 | 1 | 1 | 0 | 0 | 0 |
| 14 | 0 | 0 | 0 | 0 | 0 | 0 | 0 | 0 | 0 | 0 |
| 15 | 2 | 0 | 0 | 0 | 0 | 0 | 0 | 0 | 0 | 0 |
| 16 | 0 | 0 | 1 | 1 | 0 | 0 | 0 | 0 | 0 | 0 |
| 17 | 0 | 0 | 0 | 0 | 0 | 0 | 0 | 0 | 0 | 0 |
| 18 | 1 | 0 | 0 | 0 | 0 | 0 | 0 | 0 | 0 | 0 |
| 19 | 0 | 0 | 0 | 0 | 0 | 0 | 0 | 0 | 0 | 0 |
| 20 | 0 | 0 | 1 | 0 | 0 | 1 | 2 | 1 | 0 | 0 |
| 21 | 0 | 0 | 0 | 0 | 0 | 0 | 0 | 0 | 0 | 0 |
| 22 | 0 | 0 | 0 | 0 | 0 | 0 | 0 | 0 | 0 | 0 |
| 23 | 0 | 0 | 0 | 0 | 0 | 0 | 0 | 0 | 0 | 0 |
| 24 | 0 | 0 | 0 | 0 | 0 | 0 | 0 | 0 | 0 | 0 |
| 25 | 0 | 0 | 0 | 0 | 0 | 0 | 0 | 0 | 0 | 0 |
| 26 | 0 | 0 | 0 | 0 | 0 | 0 | 0 | 0 | 0 | 0 |
| 27 | 0 | 0 | 0 | 0 | 0 | 0 | 0 | 0 | 0 | 0 |
| 28 | 1 | 0 | 0 | 0 | 0 | 0 | 0 | 0 | 0 | 0 |
| 29 | 0 | 0 | 0 | 0 | 0 | 0 | 0 | 0 | 0 | 0 |
| 30 | 0 | 0 | 0 | 0 | 0 | 0 | 0 | 0 | 0 | 0 |
| 31 | 1 | 0 | 0 | 0 | 0 | 0 | 0 | 1 | 0 | 0 |
| 32 | 0 | 0 | 0 | 0 | 0 | 0 | 0 | 0 | 0 | 0 |
| 33 | 2 | 0 | 0 | 0 | 0 | 0 | 0 | 0 | 0 | 0 |
| 34 | 0 | 0 | 0 | 0 | 0 | 0 | 0 | 0 | 0 | 0 |
| 35 | 1 | 0 | 0 | 0 | 0 | 0 | 0 | 0 | 0 | 0 |
| 36 | 0 | 0 | 0 | 0 | 0 | 0 | 0 | 0 | 0 | 0 |
| 37 | 0 | 0 | 0 | 0 | 0 | 0 | 0 | 0 | 0 | 0 |
| 38 | 1 | 0 | 0 | 0 | 0 | 0 | 0 | 0 | 0 | 0 |
| 39 | 0 | 0 | 0 | 0 | 0 | 0 | 0 | 0 | 0 | 0 |
| 40 | 0 | 0 | 0 | 0 | 0 | 0 | 0 | 0 | 0 | 0 |
| 41 | 1 | 0 | 0 | 0 | 0 | 0 | 0 | 0 | 0 | 0 |
| 42 | 0 | 0 | 0 | 0 | 0 | 0 | 0 | 0 | 0 | 0 |
| 43 | 1 | 0 | 0 | 0 | 0 | 1 | 0 | 0 | 0 | 0 |
| 44 | 1 | 0 | 0 | 0 | 0 | 1 | 0 | 0 | 0 | 0 |
| 45 | 1 | 0 | 0 | 0 | 0 | 1 | 0 | 0 | 0 | 0 |
| 46 | 1 | 0 | 0 | 0 | 0 | 0 | 0 | 0 | 0 | 0 |
| 47 | 1 | 0 | 0 | 0 | 0 | 1 | 0 | 0 | 0 | 0 |
| 48 | 1 | 1 | 1 | 0 | 0 | 0 | 0 | 0 | 0 | 0 |
| 49 | 0 | 0 | 0 | 0 | 0 | 0 | 0 | 0 | 0 | 0 |
| 50 | 1 | 1 | 1 | 1 | 0 | 0 | 0 | 0 | 0 | 0 |
| 51 | 0 | 0 | 0 | 0 | 1 | 0 | 0 | 0 | 0 | 0 |
| 52 | 1 | 0 | 1 | 0 | 0 | 1 | 0 | 0 | 0 | 0 |
| 53 | 0 | 0 | 0 | 0 | 0 | 0 | 0 | 0 | 0 | 0 |
| 54 | 0 | 0 | 0 | 0 | 0 | 0 | 0 | 0 | 0 | 0 |
| 55 | 0 | 0 | 0 | 0 | 0 | 0 | 0 | 0 | 0 | 0 |
| 56 | 0 | 0 | 0 | 0 | 0 | 0 | 0 | 0 | 0 | 0 |
| 57 | 0 | 0 | 0 | 0 | 0 | 0 | 0 | 0 | 0 | 0 |
| 58 | 0 | 0 | 0 | 0 | 0 | 0 | 0 | 0 | 0 | 0 |
| 59 | 1 | 0 | 0 | 0 | 0 | 0 | 0 | 0 | 0 | 0 |
| 60 | 0 | 0 | 0 | 0 | 0 | 0 | 0 | 0 | 0 | 0 |
| 61 | 0 | 0 | 0 | 0 | 0 | 0 | 0 | 0 | 0 | 0 |
| 62 | 0 | 0 | 0 | 0 | 0 | 0 | 0 | 0 | 0 | 0 |
| 63 | 0 | 0 | 0 | 0 | 0 | 0 | 0 | 0 | 0 | 0 |
| 64 | 0 | 0 | 0 | 0 | 0 | 0 | 0 | 0 | 0 | 0 |
| 65 | 0 | 0 | 0 | 0 | 0 | 0 | 0 | 0 | 0 | 0 |
| 66 | 0 | 0 | 0 | 0 | 0 | 0 | 0 | 0 | 0 | 0 |
| 67 | 0 | 0 | 0 | 0 | 0 | 0 | 0 | 0 | 0 | 0 |
| 68 | 0 | 0 | 0 | 0 | 0 | 0 | 0 | 0 | 0 | 0 |
| 69 | 0 | 1 | 0 | 0 | 0 | 0 | 0 | 0 | 0 | 0 |
| 70 | 0 | 0 | 0 | 0 | 0 | 0 | 0 | 0 | 0 | 0 |
| 71 | 0 | 0 | 0 | 0 | 0 | 0 | 0 | 0 | 0 | 0 |
| 72 | 0 | 0 | 0 | 0 | 0 | 0 | 0 | 0 | 0 | 0 |
| 73 | 0 | 0 | 0 | 0 | 0 | 0 | 0 | 0 | 0 | 0 |
| 74 | 0 | 0 | 0 | 0 | 0 | 0 | 0 | 0 | 0 | 0 |
| 75 | 0 | 0 | 0 | 0 | 0 | 0 | 0 | 0 | 0 | 0 |
| 76 | 0 | 0 | 0 | 0 | 0 | 0 | 0 | 0 | 0 | 0 |
| 77 | 0 | 0 | 0 | 0 | 0 | 0 | 0 | 0 | 0 | 0 |
| 78 | 0 | 0 | 0 | 0 | 0 | 0 | 0 | 0 | 0 | 0 |
| 79 | 0 | 0 | 0 | 0 | 0 | 0 | 0 | 0 | 0 | 0 |
| 80 | 0 | 0 | 0 | 0 | 0 | 0 | 0 | 0 | 0 | 0 |
| 81 | 0 | 0 | 0 | 1 | 0 | 0 | 0 | 0 | 0 | 0 |
| 82 | 0 | 0 | 0 | 0 | 0 | 0 | 0 | 0 | 0 | 0 |
| 83 | 0 | 0 | 0 | 0 | 0 | 0 | 0 | 0 | 0 | 0 |
| 84 | 0 | 0 | 0 | 0 | 0 | 0 | 0 | 0 | 0 | 0 |
| 85 | 0 | 0 | 0 | 0 | 0 | 0 | 0 | 0 | 0 | 0 |
| 86 | 0 | 0 | 0 | 0 | 0 | 0 | 0 | 0 | 0 | 0 |
| 87 | 0 | 0 | 0 | 0 | 0 | 0 | 0 | 0 | 0 | 0 |
| 88 | 0 | 0 | 0 | 0 | 0 | 0 | 0 | 0 | 0 | 0 |
| 89 | 0 | 0 | 0 | 0 | 0 | 0 | 0 | 0 | 0 | 0 |
| 90 | 0 | 0 | 0 | 0 | 0 | 0 | 0 | 0 | 0 | 0 |
| 91 | 0 | 0 | 0 | 0 | 0 | 0 | 0 | 0 | 0 | 0 |
| 92 | 0 | 0 | 0 | 0 | 0 | 0 | 0 | 0 | 0 | 0 |
| 93 | 0 | 0 | 0 | 0 | 0 | 0 | 0 | 0 | 0 | 0 |
| 94 | 0 | 0 | 0 | 0 | 0 | 0 | 0 | 0 | 0 | 0 |
| 95 | 0 | 0 | 0 | 0 | 0 | 0 | 0 | 0 | 0 | 0 |
| 96 | 0 | 0 | 0 | 0 | 0 | 0 | 0 | 0 | 0 | 0 |
| 97 | 0 | 0 | 0 | 0 | 0 | 0 | 0 | 0 | 0 | 0 |
| 98 | 0 | 0 | 0 | 0 | 0 | 0 | 0 | 0 | 0 | 0 |
| 99 | 0 | 0 | 0 | 0 | 0 | 0 | 0 | 0 | 0 | 0 |
| 100 | 0 | 0 | 0 | 0 | 0 | 0 | 0 | 0 | 0 | 0 |
| 101 | 0 | 0 | 0 | 0 | 0 | 0 | 0 | 0 | 0 | 0 |
| 102 | 0 | 0 | 0 | 0 | 0 | 0 | 0 | 0 | 0 | 0 |

1-53: participants with Psoriatic arthritis; 55 a 102: controls

Source: The author (2023).

2 – Resistivity index of vessels detected by power doppler in the nail bed (TO BE CONTINUED)

|  | RIqd1 | RIqd2 | RIqd3 | RIqd4 | RIqd5 | RIqd6 | RIqd7 | RIqd8 | RIqd9 | RIqd10 |
| --- | --- | --- | --- | --- | --- | --- | --- | --- | --- | --- |
| 1 | . | . | . | . | . | . | . | . | . | . |
| 2 | . | . | . | . | . | . | . | . | . | . |
| 3 | 0,52 | . | . | . | . | . | . | . | . | . |
| 4 | 0,61 | . | . | . | . | . | . | . | . | . |
| 5 | . | . | . | . | . | . | . | . | . | . |
| 6 | . | . | 0,34 | . | . | 0,87 | . | . | . | . |
| 7 | . | . | . | . | . | . | . | . | . | . |
| 8 | . | . | . | . | . | . | . | . | . | . |
| 9 | 0,81 | . | . | . | . | . | . | . | . | . |
| 10 | . | . | . | . | . | . | . | . | . | . |
| 11 | . | . | . | . | . | . | . | . | . | . |
| 12 | 0,51 | . | . | . | . | 0,54 | . | . | . | . |
| 13 | . | . | . | . | . | 0,5 | 0,41 | . | . | . |
| 14 | . | . | . | . | . | . | . | . | . | . |
| 15 | 0,48 | . | . | . | . | . | . | . | . | . |
| 16 | . | . | 0,42 | 0,45 | . | . | . | . | . | . |
| 17 | . | . | . | . | . | . | . | . | . | . |
| 18 | 0,58 | . | . | . | . | . | . | . | . | . |
| 19 | 0 | . | . | . | . | . | . | . | . | . |
| 20 | . | . | . | . | . | . | . | . | . | . |
| 21 | . | . | 0,77 | . | . | 0,7 | 0,46 | 0,47 | . | . |
| 22 | . | . | . | . | . | . | . | . | . | . |
| 23 | . | . | . | . | . | . | . | . | . | . |
| 24 | . | . | . | . | . | . | . | . | . | . |
| 25 | . | . | . | . | . | . | . | . | . | . |
| 26 | . | . | . | . | . | . | . | . | . | . |
| 27 | . | . | . | . | . | . | . | . | . | . |
| 28 | . | . | . | . | . | . | . | . | . | . |
| 29 | 0,59 | . | . | . | . | . | . | . | . | . |
| 30 | . | . | . | . | . | . | . | . | . | . |
| 31 | . | . | . | . | . | . | . | . | . | . |
| 32 | 0,51 | . | . | . | . | . | . | 0,61 | . | . |
| 33 | . | . | . | . | . | . | . | . | . | . |
| 34 | 0,53 | . | . | . | . | . | . | . | . | . |
| 35 | . | . | . | . | . | . | . | . | . | . |
| 36 | 0,74 | . | . | . | . | . | . | . | . | . |
| 37 | . | . | . | . | . | . | . | . | . | . |
| 38 | . | . | . | . | . | . | . | . | . | . |
| 39 | 0,58 | . | . | . | . | . | . | . | . | . |
| 40 | . | . | . | . | . | . | . | . | . | . |
| 41 | . | . | . | . | . | . | . | . | . | . |
| 42 | 0,78 | . | . | . | . | . | . | . | . | . |
| 43 | . | . | . | . | . | . | . | . | . | . |
| 44 | . | . | . | . | . | 0,68 | . | . | . | . |
| 45 | . | . | . | . | . | 0,67 | . | . | . | . |
| 46 | 0,61 | . | . | . | . | 0,59 | . | . | . | . |
| 47 | 0,5 | . | . | . | . | . | . | . | . | . |
| 48 | 0,64 | . | . | . | . | 0,65 | . | . | . | . |
| 49 | 0,55 | 0,63 | 0,54 | . | . | . | . | . | . | . |
| 50 | . | . | . | . | . | . | . | . | . | . |
| 51 | 0,49 | . | . | 0,51 | . | . | . | . | . | . |
| 52 | . | . | . | . | . | . | . | . | . | . |
| 53 | 0,61 | . | 0,57 | . | . | 0,61 | . | . | . | . |
| 54 | . | . | . | . | . | . | . | . | . | . |
| 55 | . | . | . | . | . | . | . | . | . | . |
| 56 | . | . | . | . | . | . | . | . | . | . |
| 57 | . | . | . | . | . | . | . | . | . | . |
| 58 | . | . | . | . | . | . | . | . | . | . |
| 59 | . | . | . | . | . | . | . | . | . | . |
| 60 | 0,78 | . | . | . | . | . | . | . | . | . |
| 61 | . | . | . | . | . | . | . | . | . | . |
| 62 | . | . | . | . | . | . | . | . | . | . |
| 63 | . | . | . | . | . | . | . | . | . | . |
| 63 | . | . | . | . | . | . | . | . | . | . |
| 64 | . | . | . | . | . | . | . | . | . | . |
| 65 | . | . | . | . | . | . | . | . | . | . |
| 66 | . | . | . | . | . | . | . | . | . | . |
| 67 | . | . | . | . | . | . | . | . | . | . |
| 68 | . | . | . | . | . | . | . | . | . | . |
| 69 | . | . | . | . | . | . | . | . | . | . |
| 70 | . | . | . | . | . | . | . | . | . | . |
| 71 | . | . | . | . | . | . | . | . | . | . |
| 72 | . | . | . | . | . | . | . | . | . | . |
| 73 | . | . | . | . | . | . | . | . | . | . |
| 74 | . | . | . | . | . | . | . | . | . | . |
| 75 | . | . | . | . | . | . | . | . | . | . |
| 76 | . | . | . | . | . | . | . | . | . | . |
| 77 | . | . | . | . | . | . | . | . | . | . |
| 78 | . | . | . | . | . | . | . | . | . | . |
| 79 | . | . | . | . | . | . | . | . | . | . |
| 80 | . | . | . | . | . | . | . | . | . | . |
| 81 | . | . | . | 0,35 | . | . | . | . | . | . |
| 82 | . | . | . | . | . | . | . | . | . | . |
| 83 | . | . | . | . | . | . | . | . | . | . |
| 84 | . | . | . | . | . | . | . | . | . | . |
| 85 | . | . | . | . | . | . | . | . | . | . |
| 86 | . | . | . | . | . | . | . | . | . | . |
| 87 | . | . | . | . | . | . | . | . | . | . |
| 88 | . | . | . | . | . | . | . | . | . | . |
| 89 | . | . | . | . | . | . | . | . | . | . |
| 90 | . | . | . | . | . | . | . | . | . | . |
| 91 | . | . | . | . | . | . | . | . | . | . |
| 92 | . | . | . | . | . | . | . | . | . | . |
| 93 | . | . | . | . | . | . | . | . | . | . |
| 94 | . | . | . | . | . | . | . | . | . | . |
| 95 | . | . | . | . | . | . | . | . | . | . |
| 96 | . | . | . | . | . | . | . | . | . | . |
| 97 | . | . | . | . | . | . | . | . | . | . |
| 98 | . | . | . | . | . | . | . | . | . | . |
| 99 | . | . | . | . | . | . | . | . | . | . |
| 100 | . | . | . | . | . | . | . | . | . | . |
| 101 | . | . | . | . | . | . | . | . | . | . |
| 102 | . | . | . | . | . | . | . | . | . | . |
| 103 | . | . | . | . | . | . | . | . | . | . |
| 104 | . | . | . | . | . | . | . | . | . | . |

1-53: participants with Psoriatic arthritis; 55 a 104: controls

Note: RI = resistivity index

APPENDIX D – AssociaTION OF MDA WITH ULTRASOUND QUANTITATIVE AND CATEGORICAL VARIABLES

1 – Descriptive statistics of the variables according to the mda and p values of the statistical tests - a

| **Variable** | **MDA** | **N** | **Mean** | **Median** | **Minimum** | **Maximum** | **p*** |
| --- | --- | --- | --- | --- | --- | --- | --- |
| Number of fingers with GS score 1, 2, 3, 4 | No | 32 | 3,56 | 3 | 0 | 10 |  |
|  | Yes | 19 | 2,32 | 2 | 0 | 8 | 0,075 |
| Number of fingers with GS score 3 or 4 | No | 32 | 1,03 | 0 | 0 | 10 |  |
|  | Yes | 19 | 0,63 | 0 | 0 | 4 | 0,595 |
| Mean GS score | No | 32 | 0,73 | 0,50 | 0 | 3,70 |  |
|  | Yes | 19 | 0,46 | 0,30 | 0 | 1,80 | 0,120 |
| Mean nail bed distance | No | 32 | 1,82 | 1,84 | 1,30 | 3,17 |  |
|  | Yes | 19 | 1,75 | 1,75 | 1,28 | 2,85 | 0,179 |
| Number of fingers with bed/matrix PD > 0 | No | 32 | 1,09 | 1 | 0 | 4 |  |
|  | Yes | 19 | 0,74 | 0 | 0 | 3 | 0,370 |
| Mean bed and matrix PD | No | 32 | 0,13 | 0,10 | 0 | 0,67 |  |
|  | Yes | 19 | 0,07 | 0 | 0 | 0,30 | 0,328 |
| Number of fingers with enthesitis | No | 32 | 0,59 | 0 | 0 | 7 |  |
|  | Yes | 19 | 0,37 | 0 | 0 | 2 | 0,619 |
| Number of fingers with paratendinitis | No | 32 | 0,59 | 0 | 0 | 3 |  |
|  | Yes | 19 | 0,47 | 0 | 0 | 4 | 0,479 |
| Number of fingers with grayscale synovitis > 0 | No | 32 | 0,41 | 0 | 0 | 4 |  |
|  | Yes | 19 | 0,63 | 0 | 0 | 6 | 0,848 |
| Mean grayscale synovitis | No | 32 | 0,05 | 0 | 0 | 0,40 |  |
|  | Yes | 19 | 0,08 | 0 | 0 | 0,90 | 0,981 |
| Number of fingers with PD synovitis > 0 | No | 32 | 0 | 0 | 0 | 0 |  |
|  | Yes | 19 | 0 | 0 | 0 | 0 | - |
| Mean PD synovitis | No | 32 | 0 | 0 | 0 | 0 |  |
|  | Yes | 19 | 0 | 0 | 0 | 0 | - |
| Number of fingers with osteophytes | No | 32 | 1,97 | 1,50 | 0 | 7 |  |
|  | Yes | 19 | 1,42 | 0 | 0 | 10 | 0,188 |
| Mean score of the 10 fingers | No | 32 | 1,01 | 0,70 | 0,10 | 4,60 |  |
|  | Yes | 19 | 0,69 | 0,60 | 0 | 2,80 | 0,143 |

*Nonparametric Mann‒Whitney U test, p < 0.05

Source: The Author (2023).

2 -Descriptive statistics of the variables according to the mda and p values of the statistical tests - b

| **Variable** |  | **Classification** | **MDA** | | | | **p*** |
| --- | --- | --- | --- | --- | --- | --- | --- |
|  |  |  | No | | Yes | |  |
|  |  |  | N | % | N | % |  |
| Any fingers with GS score 3 or 4? |  | No | 2 | 6,3% | 4 | 21,1% |  |
|  |  | Yes | 30 | 93,8% | 15 | 78,9% | 0,179 |
| Any fingers with GS score > 0? |  | No | 15 | 46,9% | 11 | 57,9% |  |
|  |  | Yes | 17 | 53,1% | 8 | 42,1% | 0,565 |
| Any fingers with enthesitis? |  | No | 25 | 78,1% | 13 | 68,4% |  |
|  |  | Yes | 7 | 21,9% | 6 | 31,6% | 0,515 |
| Any fingers with paratendinitis? |  | No | 22 | 68,8% | 15 | 78,9% |  |
|  |  | Yes | 10 | 31,3% | 4 | 21,1% | 0,527 |
| Any fingers with grayscale synovitis > 0? |  | No | 22 | 68,8% | 13 | 68,4% |  |
|  |  | Yes | 10 | 31,3% | 6 | 31,6% | 1 |
| Any fingers with PD synovitis > 0? |  | No | 32 | 100% | 19 | 100% |  |
|  |  | Yes | 0 | 0% | 0 | 0% | 1 |
| Any fingers with osteophytes? |  | No | 12 | 37,5% | 10 | 52,6% |  |
|  |  | Yes | 20 | 62,5% | 9 | 47,4% | 0,383 |

*Fisher´s exact test, p<0,05

Source: The Author (2023).

APPENDIX E – AssociaTION OF DAPSA WITH QUANTITATIVE AND CATEGORICAL VARIABLES

1 – DESCRIPTIVE STATISTICS OF VARIABLES ACCORDING TO DAPSA CLASSIFICATIONS AND P-VALUES OS STATISTICAL TESTS

(TO BE CONTINUED)

| **Variable** | **DAPSA** | **n** | **Mean** | **Median** | | **Minimum** | | **Maximum** | | **p*** | |  |
| --- | --- | --- | --- | --- | --- | --- | --- | --- | --- | --- | --- | --- |
| Number of fingers with GS score 3 or 4 | Remission | 8 | 2,25 | 1 | | 0 | | 8 | |  | |  |
|  | Low activ | 29 | 2,90 | 2 | | 0 | | 10 | |  | |  |
|  | Moder activ | 10 | 4,50 | 4 | | 2 | | 9 | |  | |  |
|  | High activ | 5 | 2,60 | 2 | | 1 | | 5 | | 0,074 | |  |
|  | Remission | 8 | 0,50 | 0 | | 0 | | 3 | |  | | |
| Number of fingers with GS score >0 | Low activ | 29 | 0,86 | 0 | | 0 | | 10 | |  | | |
|  | Moder activ | 10 | 1,20 | 1 | | 0 | | 5 | |  | | |
|  | High activ | 5 | 0,80 | 1 | | 0 | | 1 | | 0,091 | | |
| Mean plate GS score | Remission | 8 | 0,48 | 0,15 | | 0 | | 1,80 | |  | | |
|  | Low activ | 29 | 0,60 | 0,33 | | 0 | | 3,70 | |  | | |
|  | Moder activ | 10 | 0,85 | 0,75 | | 0,20 | | 2,20 | |  | | |
|  | High activ | 5 | 0,52 | 0,40 | | 0,30 | | 1 | | 0,051 | | |
| Mean nail bed distance | Remission | 8 | 1,81 | 1,70 | | 1,28 | | 2,85 | |  | | |
|  | Low activ | 29 | 1,81 | 1,82 | | 1,28 | | 3,17 | |  | | |
|  | Moder activ | 10 | 1,78 | 1,81 | | 1,46 | | 2,15 | |  | | |
|  | High activ | 5 | 1,70 | 1,79 | | 1,42 | | 1,90 | | 0,930 | | |
| No. of fingers with PD > 0 | Remission | 8 | 1,13 | 0,50 | | 0 | | 3 | |  | | |
|  | Low activ | 29 | 0,79 | 0 | | 0 | | 4 | |  | | |
|  | Moder activ | 10 | 1,40 | 1 | | 0 | | 4 | |  | | |
|  | High activ | 5 | 0,80 | 1 | | 0 | | 1 | | 0,419 | | |
| Mean bed and matrix PD | Remission | 8 | 0,11 | 0,05 | | 0 | | 0,30 | |  | | |
|  | Low activ | 29 | 0,10 | 0 | | 0 | | 0,67 | |  | | |
|  | Moder activ | 10 | 0,16 | 0,15 | | 0 | | 0,40 | |  | | |
|  | High activ | 5 | 0,08 | 0,10 | | 0 | | 0,10 | | 0,400 | | |
| Number of fingers with enthesitis | Remission | 8 | 0,50 | 0 | 0 | | 2 | |  | |  |  |
|  | Low activ | 29 | 0,24 | 0 | 0 | | 2 | |  | |  |  |
|  | Moder activ | 10 | 1 | 0 | 0 | | 7 | |  | |  |  |
|  | High activ | 5 | 1 | 0 | 0 | | 4 | | 0,629 | |  |  |
| Number of fingers with paratendinitis | Remission | 8 | 0,25 | 0 | 0 | | 2 | |  | |  |  |
|  | Low activ | 29 | 0,55 | 0 | 0 | | 4 | |  | |  |  |
|  | Moder activ | 10 | 0,70 | 0 | 0 | | 3 | |  | |  |  |
|  | High activ | 5 | 0,60 | 0 | 0 | | 2 | | 0,766 | |  |  |
| Number of fingers with grayscale synovitis > 0 | Remission | 8 | 0,88 | 0 | 0 | | 6 | |  | |  |  |
|  | Low activ | 29 | 0,41 | 0 | 0 | | 2 | |  | |  |  |
|  | Moder activ | 10 | 0,20 | 0 | 0 | | 1 | |  | |  |  |
|  | High activ | 5 | 0,80 | 0 | 0 | | 4 | | 0,753 | |  |  |
| Mean grayscale synovitis | Remission | 8 | 0,13 | 0 | 0 | | 0,90 | |  | |  |  |
|  | Low activ | 29 | 0,04 | 0 | 0 | | 0,20 | |  | |  |  |
|  | Moder activ | 10 | 0,04 | 0 | 0 | | 0,20 | |  | |  |  |
|  | High activ | 5 | 0,08 | 0 | 0 | | 0,40 | | 0,892 | |  |  |
| Number of fingers with PD synovitis > 0 | Remission | 8 | 0 | 0 | 0 | | 0 | |  | |  |  |
|  | Low activ | 29 | 0 | 0 | 0 | | 0 | |  | |  |  |
|  | Moder activ | 10 | 0 | 0 | 0 | | 0 | |  | |  |  |
|  | High activ | 5 | 0 | 0 | 0 | | 0 | | - | |  |  |
| Mean PD synovitis | Remission | 8 | 0 | 0 | 0 | | 0 | |  | |  |  |
|  | Low activ | 29 | 0 | 0 | 0 | | 0 | |  | |  |  |
|  | Moder activ | 10 | 0 | 0 | 0 | | 0 | |  | |  |  |
|  | High activ | 5 | 0 | 0 | 0 | | 0 | | - | |  |  |
| Number of fingers with osteophytes | Remission | 8 | 2,13 | 1 | 0 | | 10 | |  | |  |  |
|  | Low activ | 29 | 1,76 | 1 | 0 | | 6 | |  | |  |  |
|  | Moder activ | 10 | 1,10 | 0 | 0 | | 3 | |  | |  |  |
|  | High activ | 5 | 2,60 | 1 | 0 | | 7 | | 0,646 | |  |  |
| Mean score of the 10 fingers | Remission | 8 | 0,79 | 0,55 | 0 | | 2,80 | |  | |  |  |
|  | Low activ | 29 | 0,80 | 0,60 | 0 | | 4,60 | |  | |  |  |
|  | Moder activ | 10 | 1,22 | 1,10 | 0,40 | | 2,70 | |  | |  |  |
|  | High activ | 5 | 0,84 | 0,70 | 0,40 | | 1,50 | | 0,051 | |  |  |

*Nonparametric Kruskal-Wallis test, p < 0.05

Source: The author (2023).

2 – ASSOCIATION OF DAPSA WITH ULTRASOUND CATEGORICAL VARIABLES

| **Variable** | **Classif** | **DAPSA** | | | | | | | |
| --- | --- | --- | --- | --- | --- | --- | --- | --- | --- |
|  |  | **Remission** | | **Low activity** | | **Moder activ** | | **High ativ** | |
|  |  | n | % | n | % | n | % | n | % |
| Any fingers with GS score 3 or 4? | No | 3 | 37,5% | 3 | 10,3% | 0 | 0% | 0 | 0% |
|  | Sim | 5 | 62,5% | 26 | 89,7% | 10 | 100% | 5 | 100% |
| Any fingers with GS score >0? | No | 4 | 50% | 18 | 62,1% | 3 | 30% | 1 | 20% |
|  | Sim | 4 | 50% | 11 | 37,9% | 7 | 70% | 4 | 80% |
| Any fingers with enthesitis? | No | 5 | 62,5% | 23 | 79,3% | 8 | 80% | 3 | 60% |
|  | Sim | 3 | 37,5% | 6 | 20,7% | 2 | 20% | 2 | 40% |
| Any fingers with paratendinitis? | No | 7 | 87,5% | 21 | 72,4% | 7 | 70% | 3 | 60% |
|  | Yes | 1 | 12,5% | 8 | 27,6% | 3 | 30% | 2 | 40% |
| Any fingers with grayscale synovitis > 0? | No | 6 | 75% | 18 | 62,1% | 8 | 80% | 4 | 80% |
|  | Yes | 2 | 25% | 11 | 37,9% | 2 | 20% | 1 | 20% |
| Any fingers with PD synovitis > 0? | No | 8 | 100% | 29 | 100% | 10 | 100% | 5 | 100% |
|  | Yes | 0 | 0% | 0 | 0% | 0 | 0% | 0 | 0% |
| Any fingers with osteophytes? | No | 2 | 25% | 13 | 44,8% | 6 | 60% | 1 | 20% |
|  | Yes | 6 | 75% | 16 | 55,2% | 4 | 40% | 4 | 80% |

Due to the low frequency of cases, it was not possible to apply statistical tests.

Source: The author (2023).

3- Descriptive statistics of the variables according to the dichotomous DAPSA groups

| Classification | DAPSA | | | p^*^ |
| --- | --- | --- | --- | --- |
|  | Remission or low activity, n (%) | | Moderate or high activity, n (%) |  |
| Any fingers with GS score 1, 2, 3, 4? |  |  | |  |
| No | 6 (16.2) | 0 (0) | |  |
| Yes | 31 (83.8) | 15 (100) | | 0.165 |
| Any fingers with GS score 3 or 4? |  |  | |  |
| No | 22 (59.5) | 4 (26.7) | |  |
| Yes | 15 (40.5) | 11 (73.3) | | 0.064 |
| Any fingers with enthesitis? |  |  | |  |
| No | 28 (75.7) | 11 (73.3) | |  |
| Yes | 9 (24.3) | 4 (26.7) | | 1 |
| Any fingers with paratendinitis? |  |  | |  |
| No | 28 (75.7) | 10 (66.7) | |  |
| Yes | 9 (24.3) | 5 (33.3) | | 0.511 |
| Any fingers with grayscale synovitis > 0? |  |  | |  |
| No | 24 (64.9) | 12 (80) | |  |
| Yes | 13 (35.1) | 3 (20) | | 0.340 |
| Any fingers with PD synovitis? |  |  | |  |
| No | 37 (100) | 15 (100) | |  |
| Yes | 0 (0) | 0 (0) | | 1 |
| Any fingers with osteophytes? |  |  | |  |
| No | 15 (40.5) | 7 (46.7) | |  |
| Yes | 22 (59.5) | 8 (53.3) | | 0.762 |

Notes: GS =Grayscale; PD= power doppler

^*^ Fisher's exact test, p < 0.05.

APPENDIX F – SPEARMAN CORRELATION BETWEEN METRICS AND ULTRASOUND FINDINGS

TABLE 1 - BSA AND NAIL ULTRASOUND FINDINGS

| **Variables** | **n** | **Spearman correlation coefficient** | **P** |
| --- | --- | --- | --- |
| BSA × No. of fingers with GS score 1,2,3,4 | 51 | 0,04 | 0,771 |
| BSA × No. of fingers with GS score 3 or 4 | 51 | 0,17 | 0,225 |
| BSA x Mean GS score | 51 | 0,09 | 0,536 |
| BSA x Mean nail bed distance | 51 | 0,07 | 0,610 |
| BSA x No. of fingers with PD > 0 | 51 | 0,10 | 0,465 |
| BSA x Mean bed and matrix PD | 51 | 0,14 | 0,336 |
| BSA x Number of fingers with enthesitis | 51 | 0,05 | 0,748 |
| BSA x Number of fingers with paratendinitis | 51 | 0,24 | 0,088 |
| BSA x Number of fingers with grayscale synovitis > 0 | 51 | -0,20 | 0,157 |
| BSA x Mean grayscale synovitis | 51 | -0,19 | 0,172 |
| BSA x Number of fingers with PD synovitis > 0 | 51 | - | - |
| BSA x Mean PD synovitis | 51 | - | - |
| BSA x Number of fingers with osteophytes | 51 | 0,10 | 0,467 |
| BSA x Mean score of the 10 fingers | 51 | 0,10 | 0,472 |

Source: The Author (2023).

TABLE 2 – BASDAI AND NAIL ULTRASOUND FINDINGS

| **Variables** | **n** | **Spearman correlation coefficient** | **P** |
| --- | --- | --- | --- |
| BASDAI × No. of fingers with GS score 1,2,3,4 | 18 | 0,26 | 0,297 |
| BASDAI × No. of fingers with GS score 3 or 4 | 18 | 0,01 | 0,961 |
| BASDAI x Mean GS score | 18 | 0,17 | 0,496 |
| BASDAI x Mean nail bed distance | 18 | -0,03 | 0,913 |
| BASDAI x No. of fingers with PD > 0 | 18 | 0,07 | 0,776 |
| BASDAI x Mean bed and matrix PD | 18 | 0,08 | 0,765 |
| BASDAI x Number of fingers with enthesitis | 18 | 0,16 | 0,524 |
| BASDAI x Number of fingers with paratendinitis | 18 | -0,04 | 0,861 |
| BASDAI x No. of fingers with grayscale synovitis > 0 | 18 | -0,30 | 0,234 |
| BASDAI x Mean grayscale synovitis | 18 | -0,27 | 0,288 |
| BASDAI x Number of fingers with PD synovitis > 0 | 18 | - | - |
| BASDAI x Mean PD synovitis | 18 | - | - |
| BASDAI x Number of fingers with osteophytes | 18 | 0,30 | 0,231 |
| BASDAI x Mean score of the 10 fingers | 18 | 0,15 | 0,548 |

Source: The author (2023).

**Table 3**. DAPSA and nail ultrasound findings

| **Variables** | **n** | **Spearman correlation coefficient** | **P** |
| --- | --- | --- | --- |
| DAPSA × Number of fingers with GS score 1,2,3,4 | 52 | 0,21 | 0,132 |
| **DAPSA × Number of fingers with GS score 3 or 4** | **52** | **0,30** | **0,032** |
| DAPSA x Mean GS score | 52 | 0,26 | 0,060 |
| DAPSA x Mean nail bed distance | 52 | -0,09 | 0,544 |
| DAPSA x Number of fingers with PD > 0 | 52 | 0,15 | 0,290 |
| DAPSA x Mean bed and matrix PD | 52 | 0,15 | 0,287 |
| DAPSA x Number of fingers with enthesitis | 52 | 0,08 | 0,589 |
| DAPSA x Number of fingers with paratendinitis | 52 | 0,14 | 0,333 |
| DAPSA x Number of fingers with grayscale synovitis > 0 | 52 | -0,08 | 0,577 |
| DAPSA x Mean grayscale synovitis | 52 | -0,05 | 0,706 |
| DAPSA x Number of fingers with PD synovitis > 0 | 52 | - | - |
| DAPSA x Mean PD synovitis | 52 | - | - |
| DAPSA x Number of fingers with DIP involvement | 52 | -0,05 | 0,717 |
| DAPSA x Mean score of the 10 fingers | 52 | 0,26 | 0,062 |

**Table 4**. PASI and nail ultrasound findings

| **Variables** | **N** | **Spearman correlation coefficient** | **P** |
| --- | --- | --- | --- |
| PASI × Number of fingers with GS score 1,2,3,4 | 51 | 0,07 | 0,643 |
| PASI × Number of fingers with GS score 3 or 4 | 51 | 0,14 | 0,316 |
| PASI x Mean GS score | 51 | 0,13 | 0,370 |
| PASI x Mean nail bed distance | 51 | 0,01 | 0,944 |
| PASI x Number of fingers with PD > 0 | 51 | 0,10 | 0,484 |
| PASI x Mean bed and matrix PD | 51 | 0,13 | 0,349 |
| PASI x Number of fingers with enthesitis | 51 | 0,07 | 0,646 |
| PASI x Number of fingers with paratendinitis | 51 | 0,41 | 0,003 |
| PASI x Number of fingers with grayscale synovitis > 0 | 51 | -0,15 | 0,284 |
| PASI x Mean grayscale synovitis | 51 | -0,16 | 0,249 |
| PASI x Number of fingers with PD synovitis > 0 | 51 | - | - |
| PASI x Mean PD synovitis | 51 | - | - |
| PASI x Number of fingers with DIP involvement | 51 | 0,11 | 0,428 |
| PASI x Mean score of the 10 fingers | 51 | 0,19 | 0,187 |

APPENDIX G– RESULTS OF COMPLIANCE ANALYSIS

Analysis of the agreement of the two examiners considering all 102 study participants (patients and controls) regarding having a finger with ec 1,2,3,4, PD > 0, enthesitis, paratendinitis, gray scale synovitis > 0, osteophyte (binary variables).

1. Kappa coefficients of agreement for the binary variables on US in patients with psoriatic arthritis

| Classification | Exam 1 n (%) | Exam 2 n (%) | Agreement n (%) | Kappa | 95% CI |
| --- | --- | --- | --- | --- | --- |
| Any fingers with GS score 1, 2, 3, 4? |  |  |  |  |  |
| No | 51 (50) | 58 (56.9) |  |  |  |
| Yes | 51 (50) | 44 (43.1) | 93.1 | 0.86 | 0.77–0.96 |
| Any fingers with GS score 3 or 4? |  |  |  |  |  |
| No | 73 (71.6) | 75 (73.5) |  |  |  |
| Yes | 29 (28.4) | 27 (26.5) | 98 | 0.95 | 0.88–1 |
| Any fingers with enthesitis? |  |  |  |  |  |
| No | 89 (87.3) | 92 (90.2) |  |  |  |
| Yes | 13 (12.7) | 10 (9.8) | 97.1 | 0.85 | 0.69–1 |
| Any fingers with paratendinitis? |  |  |  |  |  |
| No | 88 (86.3) | 89 (87.3) |  |  |  |
| Yes | 14 (13.7) | 13 (12.7) | 99 | 0.96 | 0.87–1 |
| Any fingers with grayscale synovitis > 0? |  |  |  |  |  |
| No | 86 (84.3) | 87 (85.3) |  |  |  |
| Yes | 16 (15.7) | 15 (14.7) | 99 | 0.96 | 0.89–1 |
| Any fingers with osteophytes? |  |  |  |  |  |
| No | 61 (59.8) | 62 (60.8) |  |  |  |
| Yes | 41 (40.2) | 40 (39.2) | 97.1 | 0.94 | 0.87–1 |

Notes: GS = grayscale.

**2.** Intraclass correlation coefficients (ICCs) for the quantitative variables related to US in psoriatic arthritis patients

| Variable | ICC^*^ | 95% CI |
| --- | --- | --- |
| d1 score | 0.920 | 0.877–0.947 |
| d2 score | 0.904 | 0.861–0.934 |
| d3 score | 0.908 | 0.868–0.937 |
| d4 score | 0.975 | 0.964–0.983 |
| d5 score | 0.960 | 0.942–0.973 |
| d6 score | 0.963 | 0.944–0.975 |
| d7 score | 0.991 | 0.987–0.994 |
| d8 score | 0.925 | 0.890–0.948 |
| d9 score | 0.947 | 0.921–0.965 |
| d10 score | 0.843 | 0.776–0.891 |
| Mean score of the 10 fingers | 0.982 | 0.967–0.989 |
| Number of fingers with GS score 1,2,3,4 | 0.947 | 0.915–0.966 |
| Number of fingers with GS score 3 or 4 | 0.974 | 0.960–0.982 |
| Mean GS score | 0.977 | 0.960–0.986 |
| Number of fingers with PD > 0 | 0.972 | 0.958–0.981 |
| Mean bed and matrix PD | 0.963 | 0.946–0.975 |
| Number of fingers with enthesitis | 0.938 | 0.909–0.957 |
| Number of fingers with paratendinitis | 0.975 | 0.963–0.983 |
| Number of toes with grayscale synovitis > 0 | 0.992 | 0.988–0.995 |
| Mean grayscale synovitis | 0.996 | 0.993–0.997 |
| Number of fingers with PD synovitis > 0 | - | - |
| Mean PD synovitis | - | - |
| Number of fingers with osteophytes | 0.995 | 0.993–0.997 |

Notes: d= digitum, GS = grayscale; PD = power doppler

^*^ Intraclass correlation coefficient and 95% confidence intervals calculated using a mixed-effects model, single measures and absolute agreement.

Interpretation: ICC < 0.05; poor reliability/ICC of 0.51–0.75; moderate reliability/ICC of 0.76–0.90; good reliability/ICC > 0.90; excellent reliability.

APPENDIX H– SCATTER DIAGRAMS : SPEARMAN

**Graphic 1** Scatter diagram: correlation between NAPSI and the number of fingers with Gray scale >=1

Legend: GS, GRAYSCALE

SOURCE: The Author

**Graphic 2** Scatter diagram : correlation between NAPSI and the number of fingers with Gray scale 3 or 4

Legend: GS, GRAYSCALE

SOURCE: The Author

**Graphic 3** Scatter diagram: correlation between NAPSI and the mean Gray scale change in the nail plate

Legend: GS, GRAYSCALE

SOURCE: The Author

**Graphic 4** Scatter diagram showing the correlation between NAPSI and mean score of the 10 fingers

SOURCE: The Author

**Graphic 5** Scatter diagram: correlation between ASDAS-CRP and the number of fingers with nail plate changes (GS) >=1

Legend: GS, GRAYSCALE

SOURCE: The Author

**Graphic 6** Scatter diagram: correlation between ASDAS-CRP and the number of fingers with grade 3 or 4 (GS) nail plate alterations

Legend: GS, GRAYSCALE

SOURCE: The Author

**Graphic 7** Scatter diagram: correlation between ASDAS-CRP and mean nail plate change (GS) score according to Wortsman

SOURCE: The Author

Each point corresponds to each patient evaluated with both ASDAS and mean grayscale (GS) scores; in this case, 14 patients had data for both variables.

**Graphic 8** Scatter diagram showing the correlation between ASDAS-CRP and the mean score of the 10 fingers

SOURCE: The Author

**Graphic 9** Scatter diagram : correlation between DAPSA and the number of fingers with grade 3 or 4 (GS) nail plate alterations

SOURCE: The Author

**Graphic 10** Scatter diagram showing the correlation between PASI and number of fingers with paratendinitis

SOURCE: The Author
